# Determining the Effect of Pterostilbene on Insulin Secretion Using Chemoproteomics

**DOI:** 10.3390/molecules25122885

**Published:** 2020-06-23

**Authors:** Chiara Cassiano, Daniela Eletto, Alessandra Tosco, Raffaele Riccio, Maria Chiara Monti, Agostino Casapullo

**Affiliations:** 1 Department of Pharmacy, University of Salerno, Via Giovanni Paolo II 132, 84084 Fisciano, Italy; ccassiano@unisa.it (C.C.); daeletto@unisa.it (D.E.); tosco@unisa.it (A.T.); riccio@unisa.it (R.R.); 2 Department of Pharmacy, University of Naples “Federico II”, Via D., Montesano 49, 80131 Naples, Italy

**Keywords:** stilbene, chemical proteomics, SNARE complex, insulin

## Abstract

Pterostilbene, the 3,5-dimethoxy derivative of resveratrol, is a well-known polyphenolic compound, mainly found in blueberries, grapevines, and *Pterocarpus marsupium* heartwood, which has recently attracted a great deal of attention due to its wide bio-pharmacological profile. Moreover, pterostilbene is more lipophilic than resveratrol, with a consequently better bioavailability and a more interesting therapeutic potential. In this work, a chemoproteomic approach, based on affinity chromatography, was applied on pterostilbene in the attempt to identify the biological targets responsible for its bioactivity. On this basis, syntaxins, a group of proteins involved in the formation of SNARE complexes mediating vesicles exocytosis, were selected among the most interesting pterostilbene interactors. In vitro and in cell assays gave evidence of the pterostilbene ability to reduce insulin secretion on glucose-stimulated pancreatic beta cells, opening the way to potential applications of pterostilbene as a supplement in the care of insulin-dependent metabolic disorders.

## 1. Introduction

**Figure 1:**
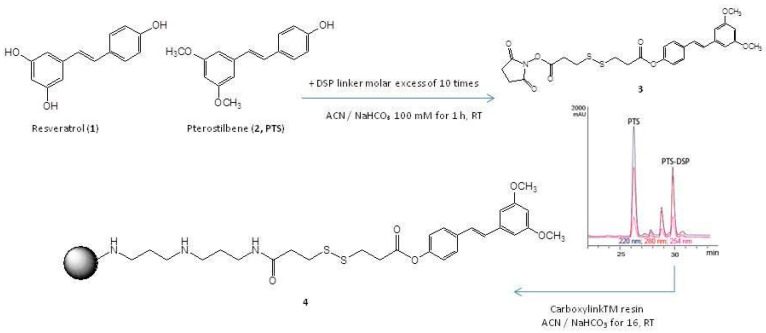
Chemical structure of resveratrol (**1**) and pterostilbene (PTS) (**2**). Reaction between PTS and the dithio-bis-succinimidyl-propionate (DSP) linker providing a covalent adduct (**3**). RP-HPLC-UV purification of 3 and reaction with Carboxylink resin, giving rise to the fishing bait (**4**).

Phytoalexins are natural compounds synthesized by plants which work as an antimicrobial defense system. Phytoalexin-containing food is well-known to exert potential human health benefits, such as cholesterol-lowering, antioxidant, anti-inflammatory, and anticancer activities [1]. Among them, stilbenes, such as resveratrol (**1**, Figure 1) and its congeners, commonly found in various dietary sources such as grapes, red wine, peanuts, and berries, gained a great attention in the last few years due to their strong antioxidant, chemo-preventive, and anti-inflammatory activities, with beneficial effects, for instance, in treating diabetes-associated macro-vascular problems [2], or neuro-protective effects [3]. Pterostilbene (trans-3,5-dimethoxy-4-hydroxystilbene, PTS, **2**, Figure 1) is one of the most interesting resveratrol derivatives, found as a main polyphenolic constituent of blueberries and the tree wood of *Pterocarpus marsupium*, used in Ayurvedic medicine for the treatment of diabetes. PTS has shifted in the last years the focus of the scientific community from resveratrol, due to its wide and interesting bioactivity spectrum and the structural peculiarities. Indeed, the presence of two dimethoxy groups, instead of the phenolic functions in resveratrol, strongly affects the lipophilicity of PTS, increasing its bioavailability, that was shown to be about 80% compared to 20% for resveratrol in an animal study [4], and making it a more suitable potential therapeutic agent. Several in vitro and in vivo pharmacological studies found the antioxidant activity of PTS strictly correlated to its therapeutic benefits against various disorders including neurodegeneration, cancer, diabetes, and vascular complications [5]. In particular, PTS was discovered to be a potent inhibitor of 5-lipoxygenase (5-LOX) activity [6], to inhibit microglial activation and production of NO and pro-inflammatory cytokines, which were also reported to drop in another in vivo study on neuro-inflammation [7]. PTS was also found to improve glycemic control [8], reduction of lipogenesis in adipose tissue and liver and induction of fatty acid oxidation in liver. Moreover, anti-carcinogenic activities in breast cancer [9] and protection against cardiovascular diseases at low concentrations (0.5–10 μM) were reported in literature [10,11,12]. In spite of its wide bioactivity profile, limited information was reported in literature about the PTS interacting macromolecular targets, and in particular the partners involved in the insulin-dependent metabolic pathways. Pancreatic β cells secrete insulin, a hormone capable to modulate plasma glucose levels. Insulin secretion and clearance are strictly regulated processes and its alteration results in the development and progression of diabetes mellitus or hyperinsulinemia syndromes [13,14].

On this basis, PTS was considered an interesting probe to be submitted to a chemoproteomic analysis, using affinity purification and mass spectrometry (AP-MS) to identify its main cellular interactors. In this approach the compound is functionalized with a reactive group that is employed for immobilization onto beads. The compound is used as a bait that, treated with cell lysates enables the enrichment of its target proteins onto the beads surface. Non-specific proteins are removed while the specific targets are recovered and identified through MS-based proteomics and SDS-PAGE/WB analysis [15,16]. With this approach, syntaxins, a group of proteins involved in insulin exocytosis, were identified as relevant PTS interactors and used as a probe to give information on the potential role of PTS in insulin-based metabolic diseases. 

## 2. Results 

The rational design of our experimental procedure consisted of three main steps: (1) PTS immobilization on a solid support; (2) identification of PTS interactors by AP in HeLa and INS-1 832/13 cell systems; (3) in vitro and in cell affinity and activity assays.

### 2.1. PTS Immobilization on a Solid Support

To achieve a quantitative immobilization of PTS on agarose beads, it was put in reaction with dithio-bis-succinimidyl-propionate (DSP), an activated disulphide-containing spacer arm, to produce a covalent adduct (**3**, Figure 1), whose formation was monitored by RP-HPLC-MS (rt of 30 min, MW = 546.1 Da) and confirmed by MSMS analysis, as deduced by the main fragment at *m/z* 431.08, compatible with the loss of *N*-hydroxysuccinimide (Figures S1 and S2). Purification of **3** by RP-HPLC-UV was followed by its immobilization on an agarose solid support (Carboxylink), through reaction between the primary amino-groups on the matrix surface and the PTS-adduct carbonyl group activated with N- hydroxysuccinimide (**4**, Figure 1), with a final 95% immobilization yield (Figure S3). All residual amino groups on the resin were then quenched with acetic anhydride. The use of a spacer arm between the small molecule and the solid support is a common practice in chemical proteomics to prevent steric hindrance that could hamper the protein accessibility, during the phase of interaction.

### 2.2. Identification of PTS Interactors by AP-MS 

**Figure 2:**
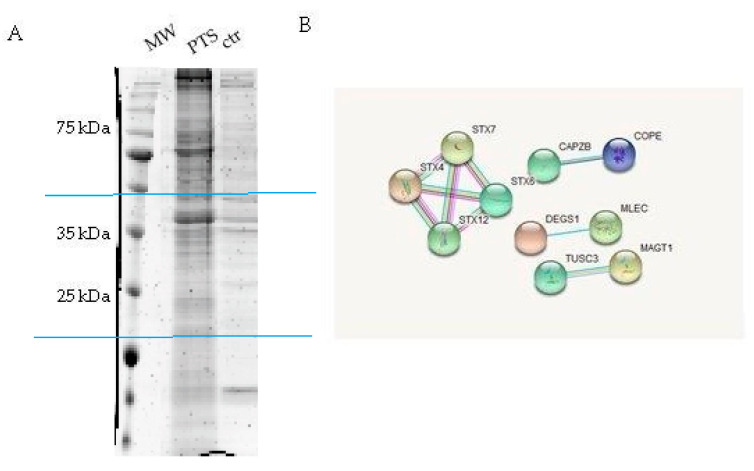
Panel **A**: SDS-PAGE of the eluted proteins from PTS-bearing and control-beads (two independent experiments); gel regions submitted to trypsin digestion included in blue lines. Panel **B**: String networks between PTS partners (https://string-db.org/).

HeLa cells protein extracts were chosen as a model system and incubated for 60 min with the PTS-modified and control beads to promote the interaction between the immobilized compound and its potential partner(s) in solution. After recovery of the solid phase, the amount of non-specific interactions was reduced by several washing steps of the matrix beads, while the tightly bound proteins were released after treatment with Laemmli buffer. The protein mixtures eluted from PTS and control experiments were resolved by 12% SDS-PAGE (Figure 2A), and the gel lanes were compared, revealing the main differences between PTS and negative (untreated matrix) control around 25 and 35 kDa: These regions were excised, divided into few pieces and subjected to an in situ digestion protocol [17]. The peptides originating from each gel slice were analyzed through nano-flow RP-HPLC MS/MS and proteins identification was performed by submitting the MS peak lists to Mascot database (Figures S4 and S5). The PTS interacting proteins list was then refined by removing the hits shared with the control experiments. The comparative combination of two independent experiments gave a final confident large list of putative PTS interactors. Due to the pleiotropic action of PTS in many different field of pharmacology, it seems reasonable that this molecule is a multi-target-directed ligand with a wide spectrum of interaction. Indeed, many proteins were identified as possible targets and among them, we were intrigued by the syntaxins family (Figure 2B and Table 1). 

They all belong to a set of proteins involved in the formation of the so called SNARE (Soluble NSF Attachment protein REceptor, where NSF stands for *N*-ethyl-maleimide-Sensitive Fusion protein) complexes, in which syntaxins act together with synaptobrevin and SNAP-25 proteins. These proteins form a tight complex both in vivo and in vitro, and their assembly is thought to be one of the key steps in vesicles exocytosis. About 30 members of the SNARE family have been found in mammalian cells, each in a distinct subcellular compartment and mediating almost all the intracellular membrane fusion events [18]. In particular, STX1 (syntaxin 1), SNAP-25 (25 kDa synaptosome-associated protein) and VAMP-2 (vesicle-associated membrane protein, also called synaptobrevin) form an extremely stable complex resistant to SDS, heat denaturation (up to around 90 °C) and protease digestion [19]. From a functional point of view, SNAP-25, STX-1, and VAMP-2 play a key role in regulating vesicles exocytosis. For instance, neuronal SNARE proteins mediate neurotransmitter release at the synapse by facilitating the fusion of vesicles to the presynaptic plasma membrane. Moreover, in beta pancreatic cells, SNARE complex seems to be involved in the early state of insulin secretion [20]. 

**Figure 3:**
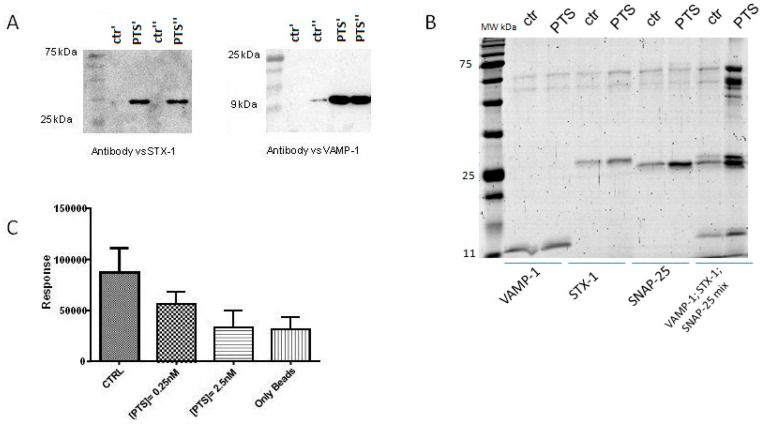
Panel **A**: Immunoblotting analysis of PTS AP experiments, performed in duplicate (ctr’/PTS’ and ctr”/PTS”) and loaded on the same SDS PAGE, showing the enrichment of STX-1 and VAMP-2 on INS-1 832/13 cell lysates. Panel **B**: In vitro PTS AP experiments carried out in duplicate on samples containing solely VAMP-2, STX-1 SNAP-25, respectively, or their 1:1:1 mixture, showing the capability of PTS to fish out the complex more than single proteins. Panel **C**: Alpha Screen results showing PTS interference with SNAP-25-VAMP-2-STX-1 complex formation, mean ± SD of four replicates is shown.

On the basis of this last evidence and the well-known PTS involvement in glucose homeostasis, we moved to perform the AP experiment on pancreatic beta-cells (INS1 832/13), trying to provide a more sound evidence of PTS interaction with the SNARE complex. Then, following the above described procedure, PTS-modified and control beads were incubated with INS-1 832/13 lysates. The PTS interacting proteins were loaded on a SDS-PAGE and revealed by immunoblotting using an antibody anti-STX1. As clearly reported in Figure 3A, PTS fished out STX-1 in INS-1 832/13 pancreatic beta-cells lysate, giving a clear proof of PTS involvement in the SNARE complex activity.

Besides, the AP experiment was carried out on four samples, three of them containing the single proteins VAMP-2, STX-1, and SNAP-25 separately and the last containing a 1:1:1 molar ratio mixture of the three proteins, using both the matrix loaded with PTS and the one without. As clearly visible from Figure 3B, PTS is almost incapable to interact with its supposed partners when they are isolated in their native unstructured conformation. On the contrary, when the proteins are previously mixed together, oligomeric complexes are formed with a different conformational state, giving rise to efficient fishing by PTS (in respect of ctrl beads) [18,21,22,23].

### 2.3. In Vitro and In Cell Assays

To further investigate the effects of PTS on the SNARE complex proteins, in vitro and in cell assays were developed.

First, an in vitro Amplified Luminescent Proximity Homogeneous Assay (Alpha Screen) was performed. In this experiment, VAMP-2 was linked to Ni^2+^ chelating donor beads through its *N*-terminal tag bearing six histidines and STX-1A was linked to Protein A-acceptor beads through an opportune antibody. Then, to promote the formation of the ternary complex, SNAP-25 was added to the reaction mixture. An AlphaScreen signal was measured due to the proximity of the mixture components allowing singlet oxygen transfer, in presence or in absence of increasing concentration of PTS (0.25 e 2.5 nM). As reported in Figure 3C and Figure S6, a relevant inhibition of the ternary complex formation was evident, after treating the mixture with PTS, in a concentration dependent manner.

A control experiment was also performed to rule out the possibility that PTS, as potential oxygen scavenger, could interfere with AlphaScreen assay quenching singlet oxygen and reduce the assay signal in a mechanism-independent manner. To this end, GST-Acceptor beads, Streptavin-Donor beads and a GST-Biotin bivalent ligand were incubated in presence and in absence of PTS. As shown in Figure S5, PTS was unable to perturb this system, even at μM concentration.

**Figure 4:**
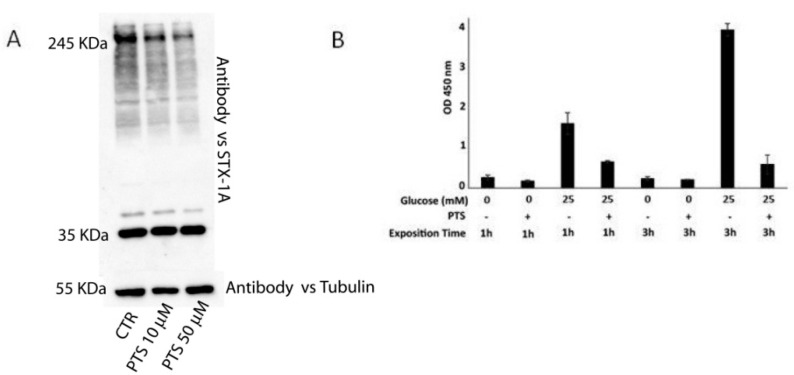
Panel **A**: Immunoblotting analysis of INS-1 832/13 cell lysates, obtained after PTS 6 h pre-incubation on living cells. Panel **B**: Insulin secretion measured by ELISA assays on INS-1 832/13 cells stimulated by glucose in presence or in absence of PTS, mean ± SD of three replicates is shown.

Then, a MTT cell viability assay was carried out to evaluate PTS cytotoxic effects on INS-1 832/13 cells. As shown in Figure S7, after 24 h of incubation, PTS is cytotoxic at concentrations ≥ 50 µM. Thus, INS-1 832/13 cells were incubated with PTS non-cytotoxic concentrations (10 and 50 µM) for 6 h, and then gently lysed using *n*-octyl-β-d-glucopiranoside, as a mild detergent useful for membrane proteins extraction without affecting the macromolecular complexes stability [21]. All samples were loaded on an 8% acrylammide SDS PAGE and revealed by Western blotting using the anti-STX-1A antibody. As clearly visible from Figure 4A, increasing concentrations of PTS strongly reduced the band intensity of high mass complexes containing STX-1A, confirming the ability of PTS to interfere with the SNARE complexes. Indeed, VAMP-2, STX-1 and SNAP-25 proteins form SNARE complexes at different oligomeric states between 60 and >200 kDa which are SDS resistant, as reported in literature [21,22,23]. 

SNARE complexes are involved in pancreatic beta cells insulin secretion [24]. Therefore, in order to evaluate the activity of PTS on this event, the amount of insulin released was compared in different conditions of glucose stimulation, in presence and absence of PTS. Insulin discharge from pancreatic islet is associated with the glucose internalization; indeed, the increase of intracellular glucose concentration induces an alteration of ATP/ADP ratio levels, favoring the blockage of ATP-dependent potassium channels, cellular depolarization, and increasing calcium ion intake through voltage-dependent calcium channels. The latter effect promotes formation of the SNARE complex and therefore, secretion of insulin [25]. 

On this basis, PTS was tested on INS-1 832/13 cells to evaluate its effect on insulin secretion. The cells were grown for 18 h in a standard culture medium and then treated with PTS at 50 μM for 3 h. Insulin secretion was then stimulated with 25 mM glucose, for 1 h and 3 h, and measured in cell secretomes using an ELISA assay [26]. In Figure 4B, the optical density values measured at 450 nm, relative to insuline amounts, clearly revealed the PTS ability to reduce insulin secretion, especially after a long glucose stimulation. 

## 3. Discussion 

The natural polyphenol pterostilbene (PTS) has been subjected to a chemoproteomic analysis, by means of affinity purification and mass spectrometry (AP-MS) approach, to outline its interactome. PTS has been firstly immobilized on agarose resin beads through a spacer arm by reaction of its free phenol moiety. The PTS-bearing solid system has then been incubated with a cell lysate in order to fish out the specific interacting proteins. Their identification by a classical bottom-up proteomic approach, consisting of 1D-SDS gel electrophoresis, trypsin digestion, mass spectrometric, and bioinformatic analysis, led to a large list of potential PTS partners, and among them syntaxins were identified as the most relevant. Syntaxins are a group of proteins involved in the SNARE complex, a multiprotein system comprising synaptobrevin (VAMP-2) and SNAP-25, mediating vesicle exocytosis processes, such as insulin secretion. On the basis of this outcome, PTS was also tested by AP and western blotting on pancreatic beta-cells (INS1 832/13), giving additional evidence of its interaction with syntaxin 1. Moreover, an in vitro AP test showed that PTS is able to fish out the oligomeric STX-1/SNAP-25/VAMP-2 complexes. 

**Figure 5:**
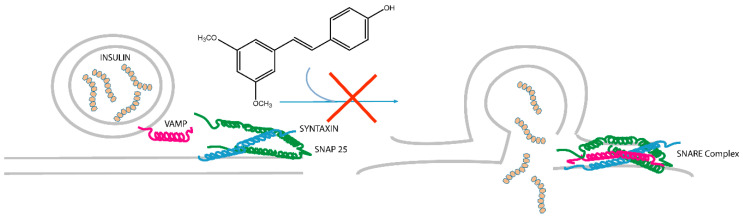
A schematic representation of PTS effect on the SNARE (Soluble NSF Attachment protein REceptor) complex assembly. PTS hampers (red cross) the assembly of STX-1/SNAP-25/ VAMP-2 (vesicle-associated membrane protein, also called synaptobrevin) ternary complex and the consequent insulin secretion.

A more convincing and rational view of the involvement of PTS in the interaction with the SNARE complex and modulation of insulin exocytosis have been gained through a set of in vitro and in cell assays. On a first instance, an ALPHA screen test gave evidence of the PTS ability in inhibiting the STX-1/SNAP-25/VAMP-2 ternary complex assembly, in a concentration-dependent trend. A similar tendency was shown treating INS-1 cells with PTS and then pointing out by 1D-SDS PAGE the lowering of high mass complexes containing STX-1A (Figure 5). As a final result, a decrease of insulin secretion was measured through ELISA test on the secretome of INS-1 cells treated with PTS and stimulated with glucose. This evidence has a relevant outcome from the bio-pharmacological point of view, since the modulation of insulin secretion could counteract the negative effects of hyperinsulinemic syndromes. Several studies demonstrated the onset of hyperinsulinemia in patients affected by metabolic syndrome [27,28,29]. On this basis, PTS could be used as pure active principle or in its functional food preparations to assist the therapy of insulin-dependent metabolic disorders.

## 4. Materials and Methods 

### 4.1. PTS Immobilization on a Functional Matrix

Formation of the PTS-DSP adduct was achieved by treating PTS with dithiobis (succinimidylpropionate), known as Lomant reagent, in molar ratio of 1:10. The reaction gave the mono-substituted adduct with a 50% yield. The reaction was carried out on 600 μg (2.34 μmoles) of PTS, solubilized in 100 μL of 100 mM ACN / NaHCO₃ and 9.5 mg (23.4 μmoles) of DSP solubilized in 200 μL of 100 mM ACN/NaHCO₃ for 1 h at rt under stirring. The reaction was monitored by Agilent 1100 HPLC using a Phenomenex column (Jupiter C4 5 µm 150 × 2.0 mm) at a flow rate of 0.2 mL/min, setting the buffer gradient B from 5% to 95% (buffer A: 100% H_2_O + 0.1% TFA; buffer B: 95% CH_3_CN + 5% H_2_O + 0.07% TFA) in 30 min. The reaction product was identified by means of a Q-TOF mass spectrometer with ESI source (Waters, Milford, MA, USA). Subsequently, the compound purification was obtained by HPLC on a Phenomenex column (Jupiter C4 5 µm 150 × 4.60 mm) at a flow rate of 1 mL/min. The immobilization was carried out by adding 500 μL of CarboxylinkTM resin to 600 μg (0.8 μmol) of the PTERO-DSP compound solubilized in 1 mL of 100 mM ACN / NaHCO_3_ 100 mM. At the same time, a control resin was prepared adding 0.8 μmol of DSP on the resin without the natural compound. The yield was estimated by superimposing the profile of the free molecule (10 μg) obtained by HPLC at 0 and 16 h on Phenomenex column (Jupiter C18 5 µm 150 × 2.00 mm) and it has been found to exceed 90%. After removing the supernatant from the resins and appropriate washing by ACN and NaHCO_3_, the DSP reactive carboxylic groups on the control resin were deactivated using 1.6 μL of ethanolamine in ACN/NaHCO_3_ 100 mM. Moreover, the residual free-amine groups on the resin samples were inactivated adding 300 mM acetic anhydride solubilized in 1 mL DMF containing 10% TEA.

### 4.2. Affinity Chromatography

HeLa and INS-1 832/13 cell lysis: HeLa cells pellets were treated with 500 μL of PBS (NaCl 137 mM, KCl 2.7 mM, Na_2_HPO_4_ 10 mM, KH_2_PO_4_ 2 mM) containing 0.1% IGEPAL to facilitate cell lysis. To avoid protein degradation, the above solution was treated with a mixture of protease inhibitors (Sigma-Aldrich, St. Louis, MI, USA: AESBF (4-(2-aminoethyl-benzensulfonyl fluoride hydrochloride), aprotinin, bestatin hydrochloride, E-64 (*N*-(trans-epoxysuccinyl)-l-leucine, 4-guanidiobutylamide, EDTA and hemisulfonated leupeptine salt). In order to allow solubilization of cell membranes favoring the release of proteins, a homogenizer was used, and the resulting solution was centrifuged for 5 min at 9390 rpm to remove debris and insoluble membranes. The concentration of the protein solution was finally estimated by Bradford and diluted to obtain a 1 mg/mL final concentration. The PTS-containing and the control resin were separately incubated with a solution containing 5 mg of protein, for 1 h under stirring at T = 4 °C. After the incubation period, 10 washings with PBS were performed to reduce the protein content unspecifically adsorbed to the support. 

Subsequently, bound proteins were eluted with 50 μL of 100 mM Tris-(2-hydroxymethyl) aminomethane (TRIS) pH 6.8, 4% (*v/v*) sodium dodecyl sulfate (SDS), 0.2% (*v/v*) Blue Bromophenol, 20% (*v/v*) glycerol and 2% β mercaptoethanol buffer. Each resin was then boiled at 95 °C in Termomixer Eppendorf for 5 min. Subsequently, 15 μL of the obtained eluates were subjected to electrophoresis on 12% polyacrylamide gel. As a reference, 10 μg of protein lysate were also loaded. The resulting gel was then treated with 40% MeOH, 10% CH_3_COOH and 50% H_2_O, then subjected to Colloidal Coating (GelCode ™ Blue Stain Reagent, Thermo Fischer Scientific, Waltham, MA, USA).

### 4.3. Protein Identification by LCMSMS Analysis 

SDS gel bands were cut in pieces and a tryptic digestion protocol in situ was performed [13]. According to the protocol, gel bands were washed with ultra-pure water and CH_3_CN treated with 1,4-dithiothreitol (DTT) for 60 min at 60 °C and with 54 mM iodoacetamide (AMBIC 50 mM) in the dark for 30 min. After several washing cycles with ACN, the samples were digested with a trypsin/LysC (Promega) 12 ng/mL for 1 h on ice. The excess of proteolytic enzyme was eliminated, and each sample was incubated overnight with 30 μL of AMBIC 50 mM at 37 °C on Termomixer Eppendorf. The supernatants were withdrawn, and the remaining peptides were extracted from the gel by 2 washings with 100% ACN. They were subsequently dried in Concentrator plus (Eppendorf, Hamburg, Germany) and resuspended in 12 μL of 10% formic Acid to perform the nano-ESI-LCMS/MS spectrometric analysis. To this end, a nanoflow liquid chromatographer (Acquity LC system; Waters Corp. Manchester, UK) equipped with a BEH C-18 column 1.7 μm, 75 μm × 250 mm (Waters Corp. Manchester, UK) was used. Five μL of each sample were then injected and separated at flow rates of 280 nL/min and with a linear gradient from 15% to 40% of buffer B (buffer A: 95% H_2_O, 5% ACN, 0.1% AA, buffer B: 95% ACN, 5% H_2_O, 0.1% AA) for 55 min. The LC-MS/MS data was acquired by means of a hybrid LTQ-ORBITRAP (Thermo Fisher Scientific, Waltham, MA, USA) spectrometer. The software used by the machine to process the data is Xcalibur, which selects double, triple, and quadruple intense ions, causing fragmentation to generate a peak list of MS / MS data. These are used to search Swiss-Prot database (release 2017_01, 553474 sequences, 198069095 residues), thanks to the Mascot server, applying the following parameters: Carbamido-methylation (C) as a fixed modification, oxidation (M) and phosphorylation (ST) as variable modifications; 30 ppm peptide tolerance; tolerance MS/MS 0.8.

### 4.4. Immunoblotting Analysis 

PTS fished out proteins from HeLa and INS-1 823/13 cell lysates were separated on a 15% polyacrylamide gel by loading 15 μL of eluates coming from two distinct replicates (0.45 μm 200 mm; Amersham, Little Chalfont, UK). In the immunoblotting experiment, the membrane was incubated for 1 h in a blocking solution containing Tris 25 mM pH 8, 125 mM NaCl, 0.05% Tween-20 (TBS-Tween), 5% milk and then exposed to the antibody primary anti-STX-1A 1:1000 (1B11-11A8; Novus Biologicals) overnight at 4 °C. After performing three washings in TBS-Tween, the membrane was incubated for 1 h at room temperature under stirring, with a secondary anti-mouse peroxidase-conjugated antibody (1:5000; Sigma Aldrich, St. Louis, MI, USA). The excess of non-specifically bound secondary antibody was then eliminated by several washings in TBS-Tween and the protein bands were exposed by exposing the membrane to an ECL solution and using a chemo-luminescence detector (LAS4000, GE Healthcare, Uppsala, Sweden).

### 4.5. Affinity Purification of Recombinant Proteins 

PTS-containing and the control resins, obtained as previously described, were divided in four aliquots and separately incubated with 100 µL of PBS containing 1 µM VAMP-2 or 1 µM STX-1A or 1 µM SNAP-25 (all the proteins were recombinant ones from Novus Biological) or a combination of the three proteins. After 30 min at 4 °C, unbounds proteins were discarded and solids supports were extensively washed with PBS. Finally, resins were treated with a denaturing buffer (Laemmli buffer) and boiled at 95 °C. Supernatants were collected and the same volume (10 µL) was loaded on 15% SDS-PAGE. Proteins were than stained with coomassie stain (GelCode™ Blue Stain Reagent, Thermo Fisher Scientific, Waltham, MA, USA)

### 4.6. Amplified Luminescent Proximity Homogeneous Assay 

During the Alpha Screen assay, colloidal dispersion of the beads and solutions of each component were made using a saline buffer containing 20 mM TRIS pH 8, 100 mM NaCl, 1 mM EDTA (Buffer A). The anti-STX-1A antibody (1B11-11A8; Novus Biologicals, Centennial, CO, USA) was incubated at a concentration of 1 nM with a suspension of 160 μg/mL of functional-activated A-acceptor beads. After 30 min at different aliquots of the above-mentioned suspension, solutions containing 2 nM STX-1A and 0.1% DMSO or PTS at 2 nM and 20 nM were added. After 45 min, the SNAP-25 protein was added at a final concentration of 0.5 nM and the resulting mixture was left for 20 min at room temperature. At the same time, VAMP-2-HiS Tagged protein and Ni^2+^-donor beads were incubated for 30 min at a concentration of 0.5 nM and 80 μg/mL, respectively. The donor and acceptor beads were mixed directly into the multiwell well of 384 wells (AlphaPlateTM-384) overnight at room temperature. As a reference, acceptor beads and donor beads were loaded in the absence of the proteins of interest. For the detection of the chemoluminescence signal produced at 520–620 nm following 680 nm irradiation and the consequent single oxygen passage between donor and acceptor beads, the EnSight Multimode Plate Reade (Perkin Elmer, Waltham, MA, USA) instrument was used.

### 4.7. INS-1 832/13 Pancreatic Cell Culture

Insulin (INS)-1 832/13 cells were maintained in RPMI 1640 (Thermo Fisher Scientific, Waltham, MA, USA) with 11 mM glucose, 10% heat inactivated FBS (Atlanta Biologicals, Oakwood, GA, USA), 100 U penicillin, 100 U streptomycin, 292 mg/mL glutamine (Thermo Fisher Scientific, Waltham, MA, USA), 1 mM sodium pyruvate (Thermo Fisher Scientific, Waltham, MA, USA), and 55 mM 2-ME.

### 4.8. INS-1 832/13 Pancreatic Cell Assay

Fifteen thousand INS-1 832/13 cells/plate were transferred in a 96-well multiwell and allowed to adhere overnight in the presence of 100 μL culture medium. Subsequently, the cells were treated with the only vehicle (DMSO at 0.1%) or PTS at the following concentrations: 1 μM, 2.5 μM, 10 μM, 25 μM, 50 μM, and 100 μM. After 24 h of treatment, 10 μL of MTT (5 mg/mL) were added to each well and the formation of the formazone salts was favored for 1 h at 37 °C. The complete dissolution of these crystals was obtained by adding to each well appropriately deprived of the medium, 100 μL of DMSO. Using a MultiskanTM spectrum sprinkler (Thermo Fisher Scientific, Waltham, MA, USA), the absorbance at two 550 nm and 620 nm wavelengths has been measured. Cell viability was then calculated based on the absorption at 520 nm and using the measured value at 620 nm as reference.

### 4.9. Western Blotting on INS-1 832/13 Lysate Treated with PTS 

INS-1 cells were grown in cell culture plates using RPMI 1640 (Rosewell Park Memorial Institute 1640 Medium, Thermo Fisher Scientific, Waltham, MA, USA) culture medium (Gibco, Thermo Fisher Scientific, Waltham, MA, USA) containing 11 mM Glucose, 10% heat- inactivated fetal bovine serum (Atlanta Biologicals), 100 U penicillin, 100 U streptomycin and 292 μg/mL glutamine (Gibco, Thermo Fisher Scientific, Waltham, MA, USA), 1 mM Sodium Piruvate (Gibco, Thermo Fisher Scientific, Waltham, MA, USA) and 55 μM β-mercaptoethanol and were treated with PTS both 10 μM and 50 μM for 6 h. INS-1 pellets were treated with 50 μL of a 20 mM Hepes, 100 mM NaCl, 2 mM EDTA solution in the presence of 1% of n-octyl-β-d-glucopyranoside to promote the release of membrane proteins. In order to avoid protein degradation, a mixture of protease inhibitors (Sigma Aldrich, St. Louis, MI, USA) consisting of AEBSF (4-(2-aminoethylbenzenesulfonyl fluorohydrochloride), aprotinin, bribon hydrochloride, E-64 (*N*-(trans-epoxy succinyl)-l-leucine-4-guanidinobutylamide, EDTA, leupeptin emisulfate salt was added. Cellular lysis was favored by using 3 freeze/thaw cycles, each 1 min, using dry ice to ensure rapid freezing and subsequently samples at 37 °C. The resulting solution was then centrifuged by Micron 17 (Thermo Fisher Scientific, Waltham, MA, USA) centrifuge for 5 min at 10,000 rpm and the supernatant was added to 50 μL of buffer samples in the presence of 2% β-mercaptoethanol. Finally, to allow complete cellular solubilization, the resulting samples were sonicated by a Sonicator Vibracell (Sonics, Turin, Italy) for 2 mL 9.9 on, 9.9 off with Ampl (amplitude) 30%. 10 μL of each sample was then loaded onto 8% polyacrylamide gel, subjected to monodimensional electrophoresis and the proteins present on the gel were transferred to a nitrocellulose membrane (0.45 μm 200 mm, Amersham, Little Chalfont, UK). The obtained membrane was boiled for 3 min with running buffer 25 mM Tris, 192 mM glycine, 0.1% SDS) to ensure optimum exposure of antibody-recognizable protein sites potentially masked within the complex. Subsequently, the membrane was incubated for 1 h with a blocking solution containing Tris 25 mM pH 8, NaCl 125 mM, 0.05% Tween-20 (TBS-Tween), 5% milk and hybridized with the primary anti-STX antibody -1A and the anti-mouse secondary antibody, following the experimental procedure previously described.

### 4.10. ELISA Assays

In order to measure the amount of insulin secreted by the INS-1 cells in the absence and in the presence of PTS, an ELISA assay was performed employing the Mercodia Ultrasensitive Insulin ELISA kit consisting of a 96-well multiwell well pre-functionalized with a primary antibody directed against human insulin. Cells were plated at a confluence of 2×10v6/60 mm, two days prior the experiment. Samples were obtained by a 20-fold dilution, carried out with Krebs buffer, of the secretion obtained from INS-1 cells under different stimulation conditions. In detail, for each condition investigated, INS-1 832/13 cells were first exposed overnight to a culture medium (RPMI) containing 5 mM glucose, and subsequently exposed to 2.5 mM glucose (glucose-stimulated insulin secretion, GSIS) [30,31]. The cells were treated with PTS at a concentration of 50 uM for 3 h. Subsequently, a treatment for 1 h or 3 h was performed in the presence or absence of 25 mM glucose solubilized in Krebs buffer (15 mM Hepes, 1.2 mM MgSO_4_, 120 mM NaCl, 5 mM KCl, 1.2 mM KH_2_PO_4_, 20 mM NaHCO_3_, 0.1% BSA). At the same time, control experiments were carried out using the same experimental conditions but adding to the cells the only vehicle (DMSO 0.1%) instead of the molecule of interest. The secretome of each sample was then harvested and diluted 20 times in Krebs buffer. According to the protocol, 25 μL of each sample and 100 μL of 1× solution enzyme were loaded on the ELISA kit plate, containing the monoclonal antibody conjugated to the enzyme peroxidase, which catalyzes a redox reaction. The plate was shaken at 100 rpm for 1 h and then 5 washes were conducted with 350 μL of 1× solution wash buffer. Each sample was incubated with 50 μL of TMB substrate (3,3′, 5,5′-tetramethylbenzidine) for 30 min at room temperature. Finally, the generation of the reaction product was stopped by adding 50 μL of 0.5 M H_2_SO_4_ (stop solution). As reference, standard insulin solutions were loaded. The absorbance at 450 nm, directly proportional to the insulin concentration, was measured with a spectrophotometer (Multiskan ™ GO, Thermo Fisher Scientific, Waltham, MA, USA).

## 5. Conclusions

Pterostilbene, a well-known antioxidant metabolite found in a red sandalwood indian tree (*Pterocarpus marsupium*), in grapes and blueberries, has been submitted to a chemical proteomic analysis to identify the main targets responsible for its wide and interesting bioactivity profile. As also reported in literature [32], pterostilbene displayed a broad interactive profile, with several proteins involved in fatty acid metabolism, oxido-reductive pathway, and vesicles exocytosis (for further details see Figure S8).

Syntaxins, a group of proteins mediating vesicle exocytosis processes, were identified as the most relevant among the list of pterostilbene potential interactors and following biological investigations on pancreatic beta cells gave evidence of the pterostilbene ability to decrease insulin secretion. These findings could be helpful for potential applications of pterostilbene as an adjuvant in the care of insulin-dependent metabolic disorders.

## Figures and Tables

**Table 1:** PTS partners list (synthaxins highlighted in yellow) including Mascot scores and matches (average values from two experiments).

Accession	Mass (Da)	Average Score	Average Matches	Description
SYPL1_HUMAN	28889	90	11	Synaptophysin-like protein 1
EMD_HUMAN	29033	306	10	Emerin
STX6_HUMAN	29215	111	3	Syntaxin-6
STX7_HUMAN	29911	129	5	Syntaxin-7
CCHL_HUMAN	30981	149	7	Cytochrome c-type heme lyase
CAPZB_HUMAN	31616	67	5	F-actin-capping protein subunit beta
STX12_HUMAN	31736	129	4	Syntaxin-12
AT1B3_HUMAN	31834	157	6	NA/K-transporting ATPase subunit beta-3
VDAC2_HUMAN	32060	756	35	Voltage-dep. anion-selective channel-2
PGAM5_HUMAN	32213	108	10	Serine/threonine-protein phosphatase PGAM5
MLEC_HUMAN	32385	653	20	Malectin
MCAT_HUMAN	33264	68	5	Carnitine/acylcarnitine carrier protein
NB5R1_HUMAN	34244	164	10	NADH-cytochrome b5 reductase 1
STX4_HUMAN	34273	146	9	Syntaxin-4
TMX2_HUMAN	34358	493	22	Thioredoxin-related transmembrane -2
DHB12_HUMAN	34416	50	6	Estradiol 17-beta-dehydrogenase 12
DHRS1_HUMAN	34458	132	4	Dehydrogenase/reductase SDR family-1
COPE_HUMAN	34688	184	4	Coatomer subunit epsilon
EMC2_HUMAN	34982	394	14	ER membrane protein complex subunit 2
COQ9_HUMAN	35658	199	10	Ubiquinone biosynthesis protein COQ9
PPP6_HUMAN	35806	269	6	Ser/thr-protein phosphatase 6 catalytic sub.
ECH1_HUMAN	36136	131	4	Delta(3,5)-Delta(2,4)-dienoyl-CoA isomerase
CIA30_HUMAN	37797	96	7	Complex I intermediate-associated protein 30
DEGS1_HUMAN	38012	180	15	Sphingolipid delta(4)-desaturase DES1
SCAM1_HUMAN	38295	353	18	Secretory carrier-associated membrane- 1
MAGT1_HUMAN	38411	120	5	Magnesium transporter protein 1
SCAM3_HUMAN	38661	441	11	Secretory carrier-associated membrane- 3
LMA2L_HUMAN	39913	109	5	VIP36-like protein
TUSC3_HUMAN	39993	192	5	Tumor suppressor candidate 3

## Supplementary Materials

The following are available online, Figure S1: MSMS spectrum of the PTS-DSP adduct, Figure S2: PTS-DSP adduct stability in PBS buffer at 0 and 2 h incubation, Figure S3 RP-HPLC-UV traces at 220 nm of PTS-DSP adduct in the surnatant of the reaction mixture with the agarose solid Carboxylink, Figure S4 Lists of the PTS partners in two independent experiments along with CTR experiments, Figure S5: Mascot Search Identifications of syntaxins, Figure S6: AlphaScreeen assay: Figure S7: MTT cell viability assays, Figure S8: STRING analysis of all the putative PTS partners arranged on the basis of their biological functions.
