# Supplementary material for: Determining the Effect of Pterostilbene on Insulin Secretion Using Chemoproteomics
Source: Molecules. 2020 Jun 23;25(12):2885. doi: 10.3390/molecules25122885 (PMC7356329; doi:10.3390/molecules25122885)

Supplementary Information

Determining the Effect of Pterostilbene on Insulin Secretion using Chemoproteomics

Chiara Cassiano, Daniela Eletto, Alessandra Tosco, Raffaele Riccio, Maria Chiara Monti, Agostino Casapullo

Chemoproteomics

Figure S1: MSMS spectrum of the PTS-DSP adduct (3) at r.t. of 30 min (see Figure 1). MH^+ of PTS-DSP is 546.09 u.m.a; its main fragment at 431.08 u.m.a. corresponds to the loss of *N*-hydroxysuccinimide.

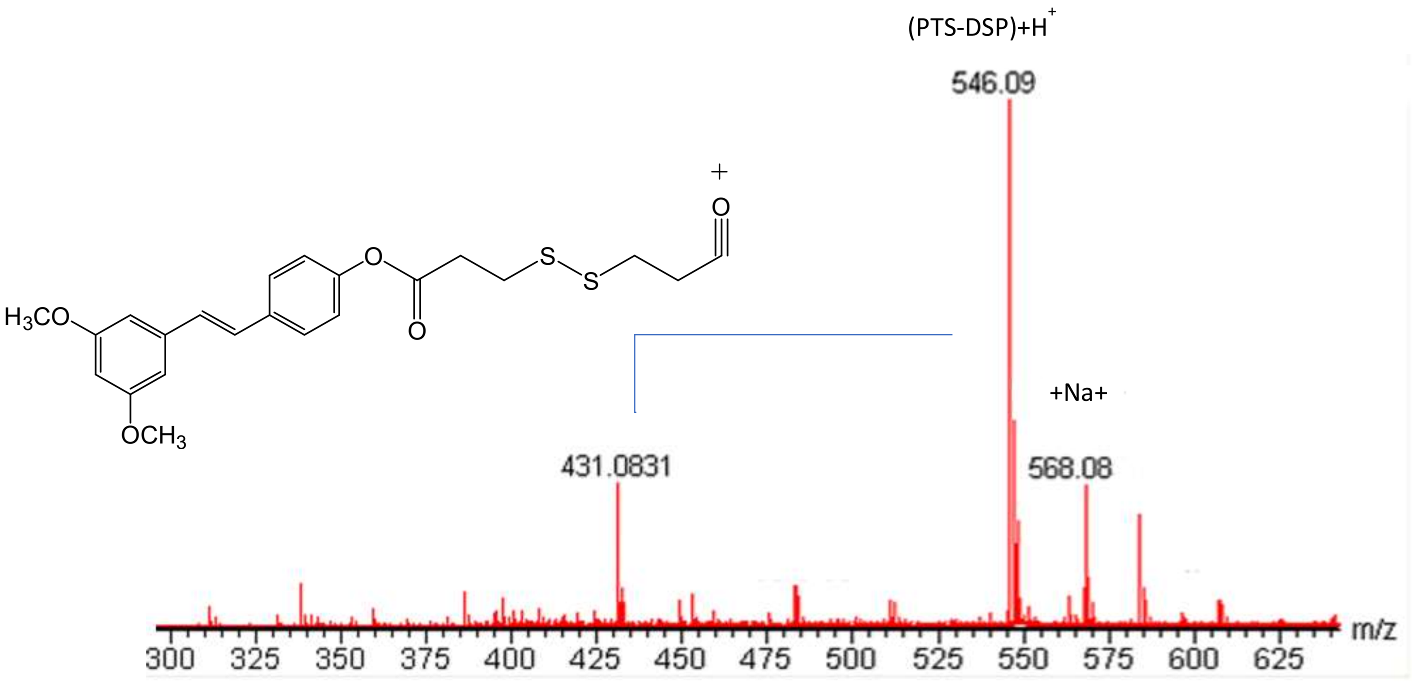

Figure S2: PTS-DSP adduct stability in PBS buffer at 0 and 2h incubation

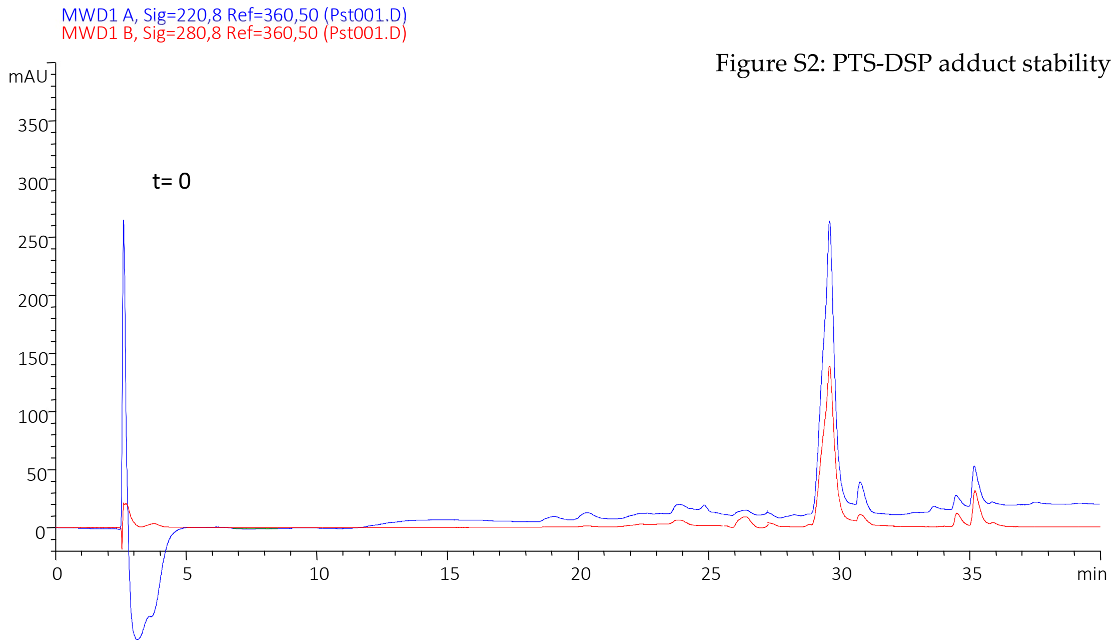

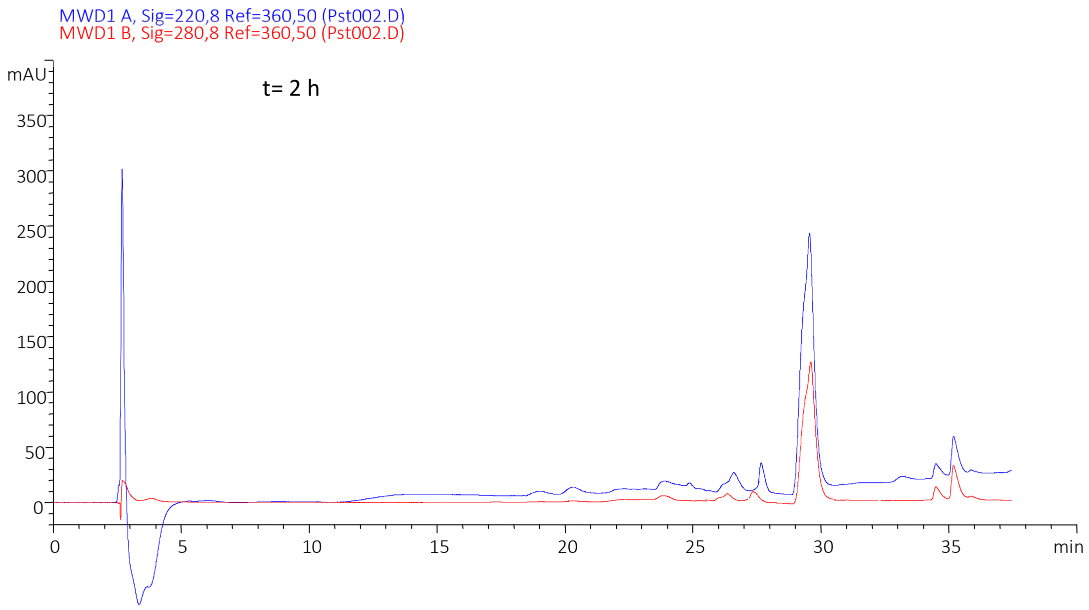

Figure S3: RP-HPLC-UV trace at 220 nm of PTS-DSP adduct in the supernatant of the reaction mixture with the agarose solid Carboxylink support at time 0 (on the left) and after 16 h of incubation at r.t. (on the right)

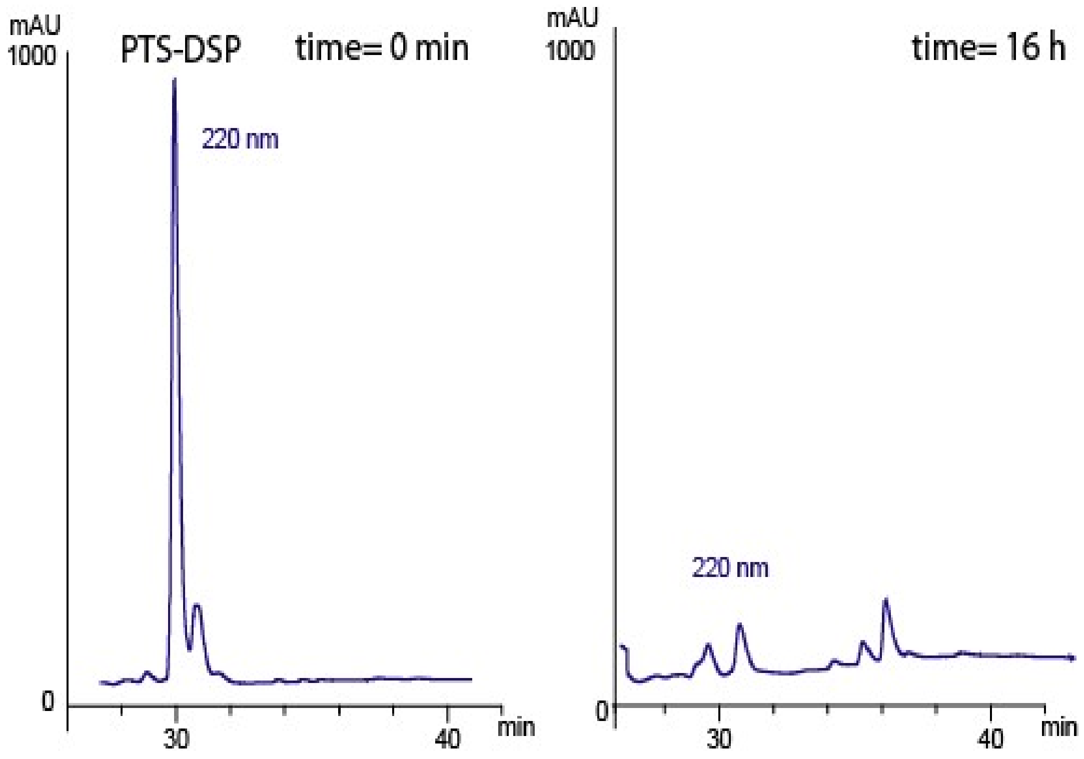

Figure S4: Lists of the PTS partners in two independent experiments along with CTR experiments.

Accession	Score	Mass	Matches	Match(sig)	Sequences	Seq(sig)	emPAI	Description
SACS_HUMAN	28	526497	22	1	12	1	0.01	Sacsin OS=Homo sapiens GN=SACS PE=1 SV=2
MYO15_HUMAN	41	397610	8	1	6	1	0.02	Unconventional myosin-XV OS=Homo sapiens GN=MYO15A PE=1 SV=2
RGPA2_HUMAN	30	213088	8	1	1	1	0.03	Ral GTPase-activating protein subunit alpha-2 OS=Homo sapiens GN=RALGAPA2 PE=1 SV=2
PHIP_HUMAN	22	208327	3	1	2	1	0.03	PH-interacting protein OS=Homo sapiens GN=PHIP PE=1 SV=2
TMX1_HUMAN	1532	32170	68	54	10	9	5.35	Thioredoxin-related transmembrane protein 1 OS=Homo sapiens GN=TMX1 PE=1 SV=1
ENPL_HUMAN	3489	92696	96	71	10	3	0.21	Endoplasmin OS=Homo sapiens GN=HSP90B1 PE=1 SV=1
VDAC2_HUMAN	774	32060	35	30	7	6	2.04	Voltage-dependent anion-selective channel protein 2 OS=Homo sapiens GN=VDAC2 PE=1 SV=2
GBB1_HUMAN	783	38151	28	23	7	6	1.55	Guanine nucleotide-binding protein G(I)/G(S)/G(T) subunit beta-1 OS=Homo sapiens GN=GNB1 PE=1 SV=3
SFXN3_HUMAN	767	36298	29	26	8	7	2.15	Sideroflexin-3 OS=Homo sapiens GN=SFXN3 PE=1 SV=2
OCAD1_HUMAN	677	27780	22	19	7	6	2.62	OCIA domain-containing protein 1 OS=Homo sapiens GN=OCIAD1 PE=1 SV=1
FOLR1_HUMAN	539	30712	20	18	6	5	1.63	Folate receptor alpha OS=Homo sapiens GN=FOLR1 PE=1 SV=3

MLEC_HUMAN	696	32385	22	18	8	6	2.01	Malectin OS=Homo sapiens GN=MLEC PE=1 SV=1
TMX2_HUMAN	442	34358	21	16	6	5	1.37	Thioredoxin-related transmembrane protein 2 OS=Homo sapiens GN=TMX2 PE=1 SV=1
SCAM1_HUMAN	444	38295	20	18	5	5	1.54	Secretory carrier-associated membrane protein 1 OS=Homo sapiens GN=SCAMP1 PE=1 SV=2
GBB4_HUMAN	381	38284	29	19	5	4	0.86	Guanine nucleotide-binding protein subunit beta-4 OS=Homo sapiens GN=GNB4 PE=1 SV=3
SSRA_HUMAN	347	32215	12	12	1	1	0.2	Translocon-associated protein subunit alpha OS=Homo sapiens GN=SSR1 PE=1 SV=3
EMC2_HUMAN	321	34982	13	12	5	5	1.34	ER membrane protein complex subunit 2 OS=Homo sapiens GN=EMC2 PE=1 SV=1
EMD_HUMAN	321	29033	11	11	4	4	1.27	Emerin OS=Homo sapiens GN=EMD PE=1 SV=1
EF1D_HUMAN	291	31217	14	11	6	5	1.6	Elongation factor 1-delta OS=Homo sapiens GN=EEF1D PE=1 SV=5
SCAM3_HUMAN	403	38661	9	8	3	3	0.59	Secretory carrier-associated membrane protein 3 OS=Homo sapiens GN=SCAMP3 PE=1 SV=3
BASI_HUMAN	255	42573	7	7	3	3	0.52	Basigin OS=Homo sapiens GN=BSG PE=1 SV=2
DHB11_HUMAN	208	33257	13	8	5	4	1.04	Estradiol 17-beta-dehydrogenase 11 OS=Homo sapiens GN=HSD17B11 PE=1 SV=3
COQ9_HUMAN	190	35658	10	7	2	2	0.65	Ubiquinone biosynthesis protein COQ9, mitochondrial OS=Homo sapiens GN=COQ9 PE=1 SV=1
VDAC3_HUMAN	187	30981	5	5	2	2	0.47	Voltage-dependent anion-selective channel protein 3 OS=Homo sapiens GN=VDAC3 PE=1 SV=1
SPEE_HUMAN	184	34373	7	6	3	3	0.68	Spermidine synthase OS=Homo sapiens GN=SRM PE=1 SV=1

TUSC3_HUMAN	228	39993	6	6	2	2	0.35	Tumor suppressor candidate 3 OS=Homo sapiens GN=TUSC3 PE=1 SV=1
ECH1_HUMAN	180	36136	4	3	3	2	0.39	Delta(3,5)-Delta(2,4)-dienoyl-CoA isomerase, mitochondrial OS=Homo sapiens GN=ECH1 PE=1 SV=2
RS3A_HUMAN	179	30154	10	6	4	3	0.81	40S ribosomal protein S3a OS=Homo sapiens GN=RPS3A PE=1 SV=2
AT1B3_HUMAN	182	31834	6	5	3	2	0.45	Sodium/potassium-transporting ATPase subunit beta-3 OS=Homo sapiens GN=ATP1B3 PE=1 SV=1
PPP6_HUMAN	170	35806	4	4	2	2	0.39	Serine/threonine-protein phosphatase 6 catalytic subunit OS=Homo sapiens GN=PPP6C PE=1 SV=1
CD166_HUMAN	264	65745	11	10	6	5	0.57	CD166 antigen OS=Homo sapiens GN=ALCAM PE=1 SV=2
LAMP2_HUMAN	85	45503	4	3	1	1	0.14	Lysosome-associated membrane glycoprotein 2 OS=Homo sapiens GN=LAMP2 PE=1 SV=2
CDK1_HUMAN	27	34131	6	1	1	1	0.19	Cyclin-dependent kinase 1 OS=Homo sapiens GN=CDK1 PE=1 SV=3
ADT2_HUMAN	37	33059	2	2	1	1	0.2	ADP/ATP translocase 2 OS=Homo sapiens GN=SLC25A5 PE=1 SV=7
TAGL3_HUMAN	24	22629	3	1	2	1	0.3	Transgelin-3 OS=Homo sapiens GN=TAGLN3 PE=1 SV=2
PORIM_HUMAN	39	21575	2	2	1	1	0.32	Porimin OS=Homo sapiens GN=TMEM123 PE=1 SV=1
MTOR_HUMAN	25	290759	10	1	5	1	0.02	Serine/threonine-protein kinase mTOR OS=Homo sapiens GN=MTOR PE=1 SV=1

UACA_HUMAN	36	163545	13	3	7	1	0.04	Uveal autoantigen with coiled-coil domains and ankyrin repeats OS=Homo sapiens GN=UACA PE=1 SV=2
XPO7_HUMAN	49	125196	6	1	5	1	0.05	Exportin-7 OS=Homo sapiens GN=XPO7 PE=1 SV=3
XPO1_HUMAN	170	124447	6	5	2	2	0.1	Exportin-1 OS=Homo sapiens GN=XPO1 PE=1 SV=1
PKHA6_HUMAN	29	117398	29	2	4	1	0.05	Pleckstrin homology domain-containing family A member 6 OS=Homo sapiens GN=PLEKHA6 PE=1 SV=4
PTPR2_HUMAN	36	112000	17	1	4	1	0.05	Receptor-type tyrosine-protein phosphatase N2 OS=Homo sapiens GN=PTPRN2 PE=1 SV=2
XPO2_HUMAN	82	111145	16	6	5	4	0.24	Exportin-2 OS=Homo sapiens GN=CSE1L PE=1 SV=3
TR150_HUMAN	22	108658	12	1	3	1	0.06	Thyroid hormone receptor-associated protein 3 OS=Homo sapiens GN=THRAP3 PE=1 SV=2
ZNT7_HUMAN	173	41941	4	4	3	3	0.53	Zinc transporter 7 OS=Homo sapiens GN=SLC30A7 PE=2 SV=1
STX4_HUMAN	163	34273	9	7	5	3	0.68	Syntaxin-4 OS=Homo sapiens GN=STX4 PE=1 SV=2
FEM1B_HUMAN	33	71189	3	1	2	1	0.09	Protein fem-1 homolog B OS=Homo sapiens GN=FEM1B PE=1 SV=1
CALX_HUMAN	56	67982	15	3	7	2	0.19	Calnexin OS=Homo sapiens GN=CANX PE=1 SV=2
BST2_HUMAN	139	20041	5	4	2	2	0.81	Bone marrow stromal antigen 2 OS=Homo sapiens GN=BST2 PE=1 SV=1
ZFP30_HUMAN	24	63456	4	1	1	1	0.1	Zinc finger protein 30 homolog OS=Homo sapiens GN=ZFP30 PE=2 SV=1

RMND1_HUMAN	58	51970	2	2	1	1	0.12	Required for meiotic nuclear division protein 1 homolog OS=Homo sapiens GN=RMND1 PE=1 SV=2
MTX1_HUMAN	126	51673	4	4	2	2	0.26	Metaxin-1 OS=Homo sapiens GN=MTX1 PE=1 SV=2
TBA4A_HUMAN	173	50634	9	5	4	3	0.42	Tubulin alpha-4A chain OS=Homo sapiens GN=TUBA4A PE=1 SV=1
TBB5_HUMAN	92	50095	6	3	4	3	0.43	Tubulin beta chain OS=Homo sapiens GN=TUBB PE=1 SV=2
COX15_HUMAN	31	46343	2	2	1	1	0.14	Cytochrome c oxidase assembly protein COX15 homolog OS=Homo sapiens GN=COX15 PE=1 SV=1
DHRS1_HUMAN	129	34458	3	2	2	1	0.19	Dehydrogenase/reductase SDR family member 1 OS=Homo sapiens GN=DHRS1 PE=1 SV=1
RS2_HUMAN	162	31590	13	10	5	3	0.76	40S ribosomal protein S2 OS=Homo sapiens GN=RPS2 PE=1 SV=2
PGTB2_HUMAN	151	37585	7	5	3	2	0.37	Geranylgeranyl transferase type-2 subunit beta OS=Homo sapiens GN=RABGGTB PE=1 SV=2
DEGS1_HUMAN	148	38012	14	8	4	3	0.6	Sphingolipid delta(4)-desaturase DES1 OS=Homo sapiens GN=DEGS1 PE=1 SV=1
PIPNB_HUMAN	145	31805	9	6	4	4	1.11	Phosphatidylinositol transfer protein beta isoform OS=Homo sapiens GN=PITPNB PE=1 SV=2
CCHL_HUMAN	136	30981	7	7	3	3	0.78	Cytochrome c-type heme lyase OS=Homo sapiens GN=HCCS PE=1 SV=1
NB5R1_HUMAN	133	34244	8	8	3	3	0.68	NADH-cytochrome b5 reductase 1 OS=Homo sapiens GN=CYB5R1 PE=1 SV=1

STX12_HUMAN	128	31736	3	3	2	2	0.45	Syntaxin-12 OS=Homo sapiens GN=STX12 PE=1 SV=1
NDUA9_HUMAN	124	42654	4	3	2	1	0.15	NADH dehydrogenase [ubiquinone] 1 alpha subcomplex subunit 9, mitochondrial OS=Homo sapiens GN=NDUFA9 PE=1 SV=2
STX7_HUMAN	116	29911	4	4	2	2	0.49	Syntaxin-7 OS=Homo sapiens GN=STX7 PE=1 SV=4
CDK4_HUMAN	122	33936	5	4	3	2	0.42	Cyclin-dependent kinase 4 OS=Homo sapiens GN=CDK4 PE=1 SV=2
MTCH1_HUMAN	114	41859	5	2	3	1	0.15	Mitochondrial carrier homolog 1 OS=Homo sapiens GN=MTCH1 PE=1 SV=1
SCAM2_HUMAN	112	37082	2	2	1	1	0.17	Secretory carrier-associated membrane protein 2 OS=Homo sapiens GN=SCAMP2 PE=1 SV=2
ELMD2_HUMAN	112	35223	7	6	2	2	0.4	ELMO domain-containing protein 2 OS=Homo sapiens GN=ELMOD2 PE=1 SV=1
COPE_HUMAN	105	34688	2	2	2	2	0.41	Coatomer subunit epsilon OS=Homo sapiens GN=COPE PE=1 SV=3
CD63_HUMAN	108	26474	5	4	2	1	0.57	CD63 antigen OS=Homo sapiens GN=CD63 PE=1 SV=2
GHC1_HUMAN	106	34904	5	3	3	2	0.41	Mitochondrial glutamate carrier 1 OS=Homo sapiens GN=SLC25A22 PE=1 SV=1
MAGT1_HUMAN	104	38411	3	3	2	2	0.36	Magnesium transporter protein 1 OS=Homo sapiens GN=MAGT1 PE=1 SV=1
STX6_HUMAN	95	29215	2	2	1	1	0.23	Syntaxin-6 OS=Homo sapiens GN=STX6 PE=1 SV=1
SGTA_HUMAN	103	34270	2	2	1	1	0.19	Small glutamine-rich tetratricopeptide repeat-containing protein alpha OS=Homo sapiens GN=SGTA PE=1 SV=1

SPG21_HUMAN	98	35223	3	2	2	1	0.18	Maspardin OS=Homo sapiens GN=SPG21 PE=1 SV=1
SYPL1_HUMAN	95	28889	8	4	2	1	0.23	Synaptophysin-like protein 1 OS=Homo sapiens GN=SYPL1 PE=1 SV=1
TM55B_HUMAN	91	30533	3	3	1	1	0.22	Type 1 phosphatidylinositol 4,5-bisphosphate 4-phosphatase OS=Homo sapiens GN=TMEM55B PE=1 SV=1
CIA30_HUMAN	88	37797	7	6	4	4	0.88	Complex I intermediate-associated protein 30, mitochondrial OS=Homo sapiens GN=NDUFAF1 PE=1 SV=2
MCU_HUMAN	89	40127	2	2	1	1	0.16	Calcium uniporter protein, mitochondrial OS=Homo sapiens GN=MCU PE=1 SV=1
TOIP1_HUMAN	86	66379	6	4	3	2	0.2	Torsin-1A-interacting protein 1 OS=Homo sapiens GN=TOR1AIP1 PE=1 SV=2
DHB7_HUMAN	86	38638	4	3	3	2	0.36	3-keto-steroid reductase OS=Homo sapiens GN=HSD17B7 PE=1 SV=1
MFF_HUMAN	86	38498	5	3	4	2	0.36	Mitochondrial fission factor OS=Homo sapiens GN=MFF PE=1 SV=1
PGAM5_HUMAN	79	32213	11	2	5	1	0.2	Serine/threonine-protein phosphatase PGAM5, mitochondrial OS=Homo sapiens GN=PGAM5 PE=1 SV=2
EPCR_HUMAN	73	26997	2	2	1	1	0.25	Endothelial protein C receptor OS=Homo sapiens GN=PROCR PE=1 SV=1
MCAT_HUMAN	67	33264	7	2	4	2	0.43	Mitochondrial carnitine/acylcarnitine carrier protein OS=Homo sapiens GN=SLC25A20 PE=1 SV=1
P5CR1_HUMAN	66	33568	1	1	1	1	0.19	Pyrroline-5-carboxylate reductase 1, mitochondrial OS=Homo sapiens GN=PYCR1 PE=1 SV=2

ODPB_HUMAN	65	39550	4	3	3	2	0.35	Pyruvate dehydrogenase E1 component subunit beta, mitochondrial OS=Homo sapiens GN=PDHB PE=1 SV=3
RDH14_HUMAN	60	37184	3	1	2	1	0.17	Retinol dehydrogenase 14 OS=Homo sapiens GN=RDH14 PE=1 SV=1
PHB_HUMAN	58	29843	2	1	2	1	0.22	Prohibitin OS=Homo sapiens GN=PHB PE=1 SV=1
MEST_HUMAN	53	38863	1	1	1	1	0.17	Mesoderm-specific transcript homolog protein OS=Homo sapiens GN=MEST PE=2 SV=2
TREX1_HUMAN	51	39525	2	2	1	1	0.16	Three-prime repair exonuclease 1 OS=Homo sapiens GN=TREX1 PE=1 SV=1
SDCB1_HUMAN	50	32595	12	3	2	1	0.2	Syntenin-1 OS=Homo sapiens GN=SDCBP PE=1 SV=1
ALG5_HUMAN	49	37150	6	3	2	2	0.38	Dolichyl-phosphate beta-glucosyltransferase OS=Homo sapiens GN=ALG5 PE=1 SV=1
S2540_HUMAN	47	38556	2	2	1	1	0.17	Solute carrier family 25 member 40 OS=Homo sapiens GN=SLC25A40 PE=2 SV=1
CAPZB_HUMAN	46	31616	6	1	2	1	0.21	F-actin-capping protein subunit beta OS=Homo sapiens GN=CAPZB PE=1 SV=4
WDR61_HUMAN	44	33731	3	2	2	1	0.19	WD repeat-containing protein 61 OS=Homo sapiens GN=WDR61 PE=1 SV=1
NSA2_HUMAN	43	30218	6	3	1	1	0.22	Ribosome biogenesis protein NSA2 homolog OS=Homo sapiens GN=NSA2 PE=1 SV=1
RS6_HUMAN	41	28834	2	1	2	1	0.23	40S ribosomal protein S6 OS=Homo sapiens GN=RPS6 PE=1 SV=1
RS27A_HUMAN	43	18296	1	1	1	1	0.38	Ubiquitin-40S ribosomal protein S27a OS=Homo sapiens GN=RPS27A PE=1 SV=2

CY1_HUMAN	36	35741	1	1	1	1	0.18	Cytochrome c1, heme protein, mitochondrial OS=Homo sapiens GN=CYC1 PE=1 SV=3
TXTP_HUMAN	34	34333	1	1	1	1	0.19	Tricarboxylate transport protein, mitochondrial OS=Homo sapiens GN=SLC25A1 PE=1 SV=2
PGES2_HUMAN	33	42088	1	1	1	1	0.15	Prostaglandin E synthase 2 OS=Homo sapiens GN=PTGES2 PE=1 SV=1
STX5_HUMAN	33	39762	1	1	1	1	0.16	Syntaxin-5 OS=Homo sapiens GN=STX5 PE=1 SV=2
VAPA_HUMAN	33	28103	2	1	2	1	0.24	Vesicle-associated membrane protein-associated protein A OS=Homo sapiens GN=VAPA PE=1 SV=3
LMA2L_HUMAN	40	39913	2	2	2	2	0.35	VIP36-like protein OS=Homo sapiens GN=LMAN2L PE=1 SV=1
P33MX_HUMAN	32	33226	9	2	3	1	0.2	Putative monooxygenase p33MONOX OS=Homo sapiens GN=KIAA1191 PE=1 SV=1
PSDE_HUMAN	31	34726	2	2	1	1	0.19	26S proteasome non-ATPase regulatory subunit 14 OS=Homo sapiens GN=PSMD14 PE=1 SV=1
DHB12_HUMAN	32	34416	3	1	2	1	0.19	Estradiol 17-beta-dehydrogenase 12 OS=Homo sapiens GN=HSD17B12 PE=1 SV=2
TSYL4_HUMAN	30	45440	10	1	3	1	0.14	Testis-specific Y-encoded-like protein 4 OS=Homo sapiens GN=TSPYL4 PE=2 SV=2
GET4_HUMAN	30	36709	1	1	1	1	0.18	Golgi to ER traffic protein 4 homolog OS=Homo sapiens GN=GET4 PE=1 SV=1
HTAI2_HUMAN	31	27260	1	1	1	1	0.24	Oxidoreductase HTATIP2 OS=Homo sapiens GN=HTATIP2 PE=1 SV=2
RLA0L_HUMAN	29	34514	3	1	2	1	0.19	60S acidic ribosomal protein P0-like OS=Homo sapiens GN=RPLP0P6 PE=5 SV=1

GLYAT_HUMAN	28	34301	2	1	2	1	0.19	Glycine N-acyltransferase OS=Homo sapiens GN=GLYAT PE=1 SV=3
TMED1_HUMAN	27	25361	2	2	1	1	0.6	Transmembrane emp24 domain-containing protein 1 OS=Homo sapiens GN=TMED1 PE=1 SV=1
MFSD3_HUMAN	26	43011	1	1	1	1	0.15	Major facilitator superfamily domain-containing protein 3 OS=Homo sapiens GN=MFSD3 PE=2 SV=1
ACTB_HUMAN	24	42052	1	1	1	1	0.15	Actin, cytoplasmic 1 OS=Homo sapiens GN=ACTB PE=1 SV=1
KDSR_HUMAN	23	36563	1	1	1	1	0.18	3-ketodihydrosphingosine reductase OS=Homo sapiens GN=KDSR PE=1 SV=1
HMOX2_HUMAN	30	36181	6	1	3	1	0.18	Heme oxygenase 2 OS=Homo sapiens GN=HMOX2 PE=1 SV=2
F177A_HUMAN	20	23799	1	1	1	1	0.28	Protein FAM177A1 OS=Homo sapiens GN=FAM177A1 PE=1 SV=1
PRAF3_HUMAN	89	21600	2	2	1	1	0.32	PRA1 family protein 3 OS=Homo sapiens GN=ARL6IP5 PE=1 SV=1
COMD8_HUMAN	15	21191	1	1	1	1	0.32	COMM domain-containing protein 8 OS=Homo sapiens GN=COMMD8 PE=1 SV=1
TCHL1_HUMAN	24	99729	7	1	1	1	0.06	Trichohyalin-like protein 1 OS=Homo sapiens GN=TCHHL1 PE=2 SV=1
MPZL1_HUMAN	20	29235	2	1	1	1	0.23	Myelin protein zero-like protein 1 OS=Homo sapiens GN=MPZL1 PE=1 SV=1

Proteins identified in PTS experiment (replicate 1)

Accession	Score	Mass	Matches	Match(sig)	Sequences	Seq(sig)	emPAI	Description
GBB1_HUMAN	948	38151	35	28	10	9	3.11	Guanine nucleotide-binding protein G(I)/G(S)/G(T) subunit beta-1 OS=Homo sapiens GN=GNB1 PE=1 SV=3
SFXN3_HUMAN	783	36298	25	24	8	8	2.75	Sideroflexin-3 OS=Homo sapiens GN=SFXN3 PE=1 SV=2
VDAC2_HUMAN	737	32060	35	28	5	5	1.54	Voltage-dependent anion-selective channel protein 2 OS=Homo sapiens GN=VDAC2 PE=1 SV=2
MLEC_HUMAN	629	32385	18	18	7	7	2.65	Malectin OS=Homo sapiens GN=MLEC PE=1 SV=1
TMX2_HUMAN	543	34358	23	19	9	7	2.38	Thioredoxin-related transmembrane protein 2 OS=Homo sapiens GN=TMX2 PE=1 SV=1
SCAM3_HUMAN	478	38661	12	12	3	3	0.59	Secretory carrier-associated membrane protein 3 OS=Homo sapiens GN=SCAMP3 PE=1 SV=3
EMC2_HUMAN	466	34982	14	14	6	6	1.8	ER membrane protein complex subunit 2 OS=Homo sapiens GN=EMC2 PE=1 SV=1
PPP6_HUMAN	368	35806	8	8	3	3	0.65	Serine/threonine-protein phosphatase 6 catalytic subunit OS=Homo sapiens GN=PPP6C PE=1 SV=1
EMD_HUMAN	291	29033	9	9	4	4	1.28	Emerin OS=Homo sapiens GN=EMD PE=1 SV=1
SCAM1_HUMAN	281	38295	15	11	5	5	1.56	Secretory carrier-associated membrane protein 1 OS=Homo sapiens GN=SCAMP1 PE=1 SV=2

COPE_HUMAN	262	34688	5	5	3	3	0.68	Coatomer subunit epsilon OS=Homo sapiens GN=COPE PE=1 SV=3
DEGS1_HUMAN	211	38012	15	11	4	4	0.88	Sphingolipid delta(4)-desaturase DES1 OS=Homo sapiens GN=DEGS1 PE=1 SV=1
COQ9_HUMAN	208	35658	9	5	2	2	0.4	Ubiquinone biosynthesis protein COQ9, mitochondrial OS=Homo sapiens GN=COQ9 PE=1 SV=1
SCAM2_HUMAN	206	37082	2	2	1	1	0.18	Secretory carrier-associated membrane protein 2 OS=Homo sapiens GN=SCAMP2 PE=1 SV=2
NB5R1_HUMAN	194	34244	12	9	5	4	1.01	NADH-cytochrome b5 reductase 1 OS=Homo sapiens GN=CYB5R1 PE=1 SV=1
LMA2L_HUMAN	178	39913	8	6	4	2	0.35	VIP36-like protein OS=Homo sapiens GN=LMAN2L PE=1 SV=1
CCHL_HUMAN	162	30981	7	6	3	3	0.79	Cytochrome c-type heme lyase OS=Homo sapiens GN=HCCS PE=1 SV=1
TUSC3_HUMAN	156	39993	4	4	2	2	0.35	Tumor suppressor candidate 3 OS=Homo sapiens GN=TUSC3 PE=1 SV=1
P5CR1_HUMAN	145	33568	2	2	1	1	0.2	Pyrroline-5-carboxylate reductase 1, mitochondrial OS=Homo sapiens GN=PYCR1 PE=1 SV=2
STX7_HUMAN	142	29911	5	5	2	2	0.49	Syntaxin-7 OS=Homo sapiens GN=STX7 PE=1 SV=4
MAGT1_HUMAN	136	38411	7	4	4	2	0.37	Magnesium transporter protein 1 OS=Homo sapiens GN=MAGT1 PE=1 SV=1
PGAM5_HUMAN	136	32213	8	5	6	3	0.75	Serine/threonine-protein phosphatase PGAM5, mitochondrial OS=Homo sapiens GN=PGAM5 PE=1 SV=2

DHRS1_HUMAN	134	34458	4	4	2	2	0.42	Dehydrogenase/reductase SDR family member 1 OS=Homo sapiens GN=DHRS1 PE=1 SV=1
GHC1_HUMAN	131	34904	2	2	1	1	0.19	Mitochondrial glutamate carrier 1 OS=Homo sapiens GN=SLC25A22 PE=1 SV=1
AT1B3_HUMAN	131	31834	5	3	3	1	0.21	Sodium/potassium-transporting ATPase subunit beta-3 OS=Homo sapiens GN=ATP1B3 PE=1 SV=1
STX12_HUMAN	130	31736	4	4	2	2	0.46	Syntaxin-12 OS=Homo sapiens GN=STX12 PE=1 SV=1
STX4_HUMAN	129	34273	9	5	3	3	0.69	Syntaxin-4 OS=Homo sapiens GN=STX4 PE=1 SV=2
MUC18_HUMAN	563	72532	32	23	9	7	1.1	Cell surface glycoprotein MUC18 OS=Homo sapiens GN=MCAM PE=1 SV=2
TKT_HUMAN	32	68519	2	1	1	1	0.09	Transketolase OS=Homo sapiens GN=TKT PE=1 SV=3
CD166_HUMAN	290	65745	16	14	8	7	0.89	CD166 antigen OS=Homo sapiens GN=ALCAM PE=1 SV=2
LAT1_HUMAN	276	55659	5	5	1	1	0.11	Large neutral amino acids transporter small subunit 1 OS=Homo sapiens GN=SLC7A5 PE=1 SV=2
GTR1_HUMAN	24	54391	1	1	1	1	0.12	Solute carrier family 2, facilitated glucose transporter member 1 OS=Homo sapiens GN=SLC2A1 PE=1 SV=2
SCG3_HUMAN	36	52973	4	1	2	1	0.12	h
CL18A_HUMAN	16	51166	4	1	1	1	0.12	C-type lectin domain family 18 member A OS=Homo sapiens GN=CLEC18A PE=2 SV=3

LAMP2_HUMAN	73	45503	2	2	1	1	0.14	Lysosome-associated membrane glycoprotein 2 OS=Homo sapiens GN=LAMP2 PE=1 SV=2
LAMP1_HUMAN	210	45367	8	8	2	2	0.3	Lysosome-associated membrane glycoprotein 1 OS=Homo sapiens GN=LAMP1 PE=1 SV=3
F71F1_HUMAN	30	39320	1	1	1	1	0.16	Protein FAM71F1 OS=Homo sapiens GN=FAM71F1 PE=2 SV=1
TCP2L_HUMAN	26	38448	2	1	1	1	0.17	Putative t-complex protein 10A homolog 2 OS=Homo sapiens GN=TCP10L2 PE=5 SV=2
ADT3_HUMAN	31	33073	3	2	2	1	0.2	ADP/ATP translocase 3 OS=Homo sapiens GN=SLC25A6 PE=1 SV=4
PORIM_HUMAN	34	21575	4	2	1	1	0.32	Porimin OS=Homo sapiens GN=TMEM123 PE=1 SV=1
RS27A_HUMAN	74	18296	4	2	2	1	0.38	Ubiquitin-40S ribosomal protein S27a OS=Homo sapiens GN=RPS27A PE=1 SV=2
ANR17_HUMAN	30	275970	7	1	5	1	0.02	Ankyrin repeat domain-containing protein 17 OS=Homo sapiens GN=ANKRD17 PE=1 SV=3
SORL_HUMAN	31	253798	6	1	5	1	0.02	Sortilin-related receptor OS=Homo sapiens GN=SORL1 PE=1 SV=2
SPG17_HUMAN	18	252781	19	1	6	1	0.02	Sperm-associated antigen 17 OS=Homo sapiens GN=SPAG17 PE=1 SV=1
PKHA6_HUMAN	35	117398	23	2	3	1	0.05	Pleckstrin homology domain-containing family A member 6 OS=Homo sapiens GN=PLEKHA6 PE=1 SV=4
TCHL1_HUMAN	24	99729	5	1	1	1	0.06	Trichohyalin-like protein 1 OS=Homo sapiens GN=TCHHL1 PE=2 SV=1

ENPL_HUMAN	2020	92696	41	37	4	2	0.14	Endoplasmin OS=Homo sapiens GN=HSP90B1 PE=1 SV=1
TOIP1_HUMAN	141	66379	3	3	2	2	0.2	Torsin-1A-interacting protein 1 OS=Homo sapiens GN=TOR1AIP1 PE=1 SV=2
PCAT2_HUMAN	31	60797	5	1	2	1	0.1	Lysophosphatidylcholine acyltransferase 2 OS=Homo sapiens GN=LPCAT2 PE=1 SV=1
MATN3_HUMAN	29	54379	1	1	1	1	0.12	Matrilin-3 OS=Homo sapiens GN=MATN3 PE=1 SV=2
MTX1_HUMAN	99	51673	4	4	2	2	0.26	Metaxin-1 OS=Homo sapiens GN=MTX1 PE=1 SV=2
TBA1B_HUMAN	110	50804	5	3	3	1	0.13	Tubulin alpha-1B chain OS=Homo sapiens GN=TUBA1B PE=1 SV=1
COX15_HUMAN	35	46343	3	2	1	1	0.14	Cytochrome c oxidase assembly protein COX15 homolog OS=Homo sapiens GN=COX15 PE=1 SV=1
DHB12_HUMAN	127	34416	8	6	4	3	0.69	Estradiol 17-beta-dehydrogenase 12 OS=Homo sapiens GN=HSD17B12 PE=1 SV=2
STX6_HUMAN	127	29215	4	4	3	3	0.85	Syntaxin-6 OS=Homo sapiens GN=STX6 PE=1 SV=1
ZNT7_HUMAN	124	41941	4	3	3	2	0.33	Zinc transporter 7 OS=Homo sapiens GN=SLC30A7 PE=2 SV=1
NDUA9_HUMAN	115	42654	6	4	3	1	0.15	NADH dehydrogenase [ubiquinone] 1 alpha subcomplex subunit 9, mitochondrial OS=Homo sapiens GN=NDUFA9 PE=1 SV=2
TM55B_HUMAN	108	30533	3	3	1	1	0.22	Type 1 phosphatidylinositol 4,5-bisphosphate 4-phosphatase OS=Homo sapiens GN=TMEM55B PE=1 SV=1

CIA30_HUMAN	104	37797	7	6	4	3	0.61	Complex I intermediate-associated protein 30, mitochondrial OS=Homo sapiens GN=NDUFAF1 PE=1 SV=2
BASI_HUMAN	93	42573	3	2	2	1	0.15	Basigin OS=Homo sapiens GN=BSG PE=1 SV=2
CAPZB_HUMAN	90	31616	6	3	2	1	0.21	F-actin-capping protein subunit beta OS=Homo sapiens GN=CAPZB PE=1 SV=4
HAX1_HUMAN	88	31601	4	3	3	2	0.46	HCLS1-associated protein X-1 OS=Homo sapiens GN=HAX1 PE=1 SV=2
SDCB1_HUMAN	86	32595	12	5	2	1	0.2	Syntenin-1 OS=Homo sapiens GN=SDCBP PE=1 SV=1
SYPL1_HUMAN	84	28889	14	4	2	1	0.23	Synaptophysin-like protein 1 OS=Homo sapiens GN=SYPL1 PE=1 SV=1
ECH1_HUMAN	81	36136	4	2	3	1	0.18	Delta(3,5)-Delta(2,4)-dienoyl-CoA isomerase, mitochondrial OS=Homo sapiens GN=ECH1 PE=1 SV=2
HMOX2_HUMAN	80	36181	7	2	3	1	0.18	Heme oxygenase 2 OS=Homo sapiens GN=HMOX2 PE=1 SV=2
SPG21_HUMAN	79	35223	2	2	1	1	0.19	Maspardin OS=Homo sapiens GN=SPG21 PE=1 SV=1
CDK1_HUMAN	77	34131	9	3	3	2	0.42	Cyclin-dependent kinase 1 OS=Homo sapiens GN=CDK1 PE=1 SV=3
CAMLG_HUMAN	76	33217	2	2	1	1	0.2	Calcium signal-modulating cyclophilin ligand OS=Homo sapiens GN=CAMLG PE=1 SV=1
ELMD2_HUMAN	71	35223	1	1	1	1	0.19	ELMO domain-containing protein 2 OS=Homo sapiens GN=ELMOD2 PE=1 SV=1

TPC_HUMAN	69	35887	5	2	3	1	0.18	Mitochondrial thiamine pyrophosphate carrier OS=Homo sapiens GN=SLC25A19 PE=1 SV=1
MCAT_HUMAN	69	33264	3	2	2	2	0.43	Mitochondrial carnitine/acylcarnitine carrier protein OS=Homo sapiens GN=SLC25A20 PE=1 SV=1
ALG5_HUMAN	67	37150	4	3	3	2	0.38	Dolichyl-phosphate beta-glucosyltransferase OS=Homo sapiens GN=ALG5 PE=1 SV=1
MPZL1_HUMAN	65	29235	5	3	3	2	0.51	Myelin protein zero-like protein 1 OS=Homo sapiens GN=MPZL1 PE=1 SV=1
MEST_HUMAN	62	38863	2	1	1	1	0.17	Mesoderm-specific transcript homolog protein OS=Homo sapiens GN=MEST PE=2 SV=2
MTCH1_HUMAN	61	41859	6	2	2	1	0.33	Mitochondrial carrier homolog 1 OS=Homo sapiens GN=MTCH1 PE=1 SV=1
PSME3_HUMAN	56	29602	2	2	1	1	0.22	Proteasome activator complex subunit 3 OS=Homo sapiens GN=PSME3 PE=1 SV=1
MSMO1_HUMAN	52	35592	2	2	1	1		Methylsterol monooxygenase 1 OS=Homo sapiens GN=MSMO1 PE=1 SV=1
TREX1_HUMAN	50	39525	2	2	1	1	0.16	Three-prime repair exonuclease 1 OS=Homo sapiens GN=TREX1 PE=1 SV=1
PP2AB_HUMAN	49	36123	4	2	2	1	0.18	Serine/threonine-protein phosphatase 2A catalytic subunit beta isoform OS=Homo sapiens GN=PPP2CB PE=1 SV=1
HTAI2_HUMAN	37	27260	1	1	1	1	0.25	Oxidoreductase HTATIP2 OS=Homo sapiens GN=HTATIP2 PE=1 SV=2
NSA2_HUMAN	36	30218	5	2	1	1	0.22	Ribosome biogenesis protein NSA2 homolog OS=Homo sapiens GN=NSA2 PE=1 SV=1

EPCR_HUMAN	34	26997	2	1	1	1	0.25	Endothelial protein C receptor OS=Homo sapiens GN=PROCR PE=1 SV=1
CDK4_HUMAN	33	33936	4	1	4	1	0.19	Cyclin-dependent kinase 4 OS=Homo sapiens GN=CDK4 PE=1 SV=2
DHB7_HUMAN	27	38638	4	2	4	2	0.36	3-keto-steroid reductase OS=Homo sapiens GN=HSD17B7 PE=1 SV=1
MFF_HUMAN	25	38498	2	1	2	1	0.17	Mitochondrial fission factor OS=Homo sapiens GN=MFF PE=1 SV=1
YIF1B_HUMAN	23	34527	1	1	1	1	0.19	Protein YIF1B OS=Homo sapiens GN=YIF1B PE=1 SV=1
WDR61_HUMAN	21	33731	3	1	2	1	0.19	WD repeat-containing protein 61 OS=Homo sapiens GN=WDR61 PE=1 SV=1

Proteins identified in PTS experiment (replicate 2)

Accession	Score	Mass	Matches	Match(sig)	Sequences	Seq(sig)	emPAI	Description
K2C1_HUMAN	3344	66170	103	96	24	22	7.82	Keratin, type II cytoskeletal 1 OS=Homo sapiens GN=KRT1 PE=1 SV=6
K22E_HUMAN	1722	65678	68	58	18	16	4.18	Keratin, type II cytoskeletal 2 epidermal OS=Homo sapiens GN=KRT2 PE=1 SV=2
K2C5_HUMAN	410	62568	16	15	7	7	0.96	Keratin, type II cytoskeletal 5 OS=Homo sapiens GN=KRT5 PE=1 SV=3
K1C10_HUMAN	2916	59020	101	75	18	16	4.08	Keratin, type I cytoskeletal 10 OS=Homo sapiens GN=KRT10 PE=1 SV=6
K1C14_HUMAN	732	51872	27	22	9	8	1.52	Keratin, type I cytoskeletal 14 OS=Homo sapiens GN=KRT14 PE=1 SV=4

K1C16_HUMAN	543	51578	23	18	8	6	1.01	Keratin, type I cytoskeletal 16 OS=Homo sapiens GN=KRT16 PE=1 SV=4
K1C9_HUMAN	1245	62255	51	46	13	12	2.5	Keratin, type I cytoskeletal 9 OS=Homo sapiens GN=KRT9 PE=1 SV=3
OXDD_HUMAN	259	38024	135	23	1	1	0.17	D-aspartate oxidase OS=Homo sapiens GN=DDO PE=2 SV=1
ATPG_HUMAN	197	33032	8	6	5	3	0.72	ATP synthase subunit gamma, mitochondrial OS=Homo sapiens GN=ATP5C1 PE=1 SV=1
RDH11_HUMAN	182	35763	11	5	5	2	0.4	Retinol dehydrogenase 11 OS=Homo sapiens GN=RDH11 PE=1 SV=2
MPCP_HUMAN	123	40525	5	5	2	2	0.34	Phosphate carrier protein, mitochondrial OS=Homo sapiens GN=SLC25A3 PE=1 SV=2
VDAC1_HUMAN	106	30868	6	4	3	2	0.47	Voltage-dependent anion-selective channel protein 1 OS=Homo sapiens GN=VDAC1 PE=1 SV=2
LMAN2_HUMAN	102	40545	8	4	5	2	0.34	Vesicular integral-membrane protein VIP36 OS=Homo sapiens GN=LMAN2 PE=1 SV=1
RS3_HUMAN	101	26842	5	3	3	2	0.56	40S ribosomal protein S3 OS=Homo sapiens GN=RPS3 PE=1 SV=2
ADT2_HUMAN	96	33059	5	5	3	3	0.72	ADP/ATP translocase 2 OS=Homo sapiens GN=SLC25A5 PE=1 SV=7
LRC59_HUMAN	91	35308	4	4	3	3	0.66	Leucine-rich repeat-containing protein 59 OS=Homo sapiens GN=LRRC59 PE=1 SV=1
PHB2_HUMAN	88	33276	7	3	5	2	0.43	Prohibitin-2 OS=Homo sapiens GN=PHB2 PE=1 SV=2
NB5R3_HUMAN	82	34441	6	3	4	2	0.42	NADH-cytochrome b5 reductase 3 OS=Homo sapiens GN=CYB5R3 PE=1 SV=3

GBLP_HUMAN	70	35511	6	4	2	2	0.4	Guanine nucleotide-binding protein subunit beta-2-like 1 OS=Homo sapiens GN=GNB2L1 PE=1 SV=3
IPO5_HUMAN	69	125032	2	2	1	1	0.05	Importin-5 OS=Homo sapiens GN=IPO5 PE=1 SV=4
DCD_HUMAN	65	11391	2	2	1	1	0.68	Dermcidin OS=Homo sapiens GN=DCD PE=1 SV=2
SFXN1_HUMAN	54	35881	5	2	4	2	0.4	Sideroflexin-1 OS=Homo sapiens GN=SFXN1 PE=1 SV=4
PPT1_HUMAN	53	34627	1	1	1	1	0.19	Palmitoyl-protein thioesterase 1 OS=Homo sapiens GN=PPT1 PE=1 SV=1
DIRA2_HUMAN	43	22813	1	1	1	1	0.3	GTP-binding protein Di-Ras2 OS=Homo sapiens GN=DIRAS2 PE=1 SV=1
BACH2_HUMAN	38	94644	21	5	1	1	0.07	Transcription regulator protein BACH2 OS=Homo sapiens GN=BACH2 PE=1 SV=1
SNAA_HUMAN	38	33667	1	1	1	1	0.19	Alpha-soluble NSF attachment protein OS=Homo sapiens GN=NAPA PE=1 SV=3
ZN326_HUMAN	38	65955	8	1	1	1	0.1	DBIRD complex subunit ZNF326 OS=Homo sapiens GN=ZNF326 PE=1 SV=2
GBB2_HUMAN	38	38048	5	2	2	2	0.37	Guanine nucleotide-binding protein G(I)/G(S)/G(T) subunit beta-2 OS=Homo sapiens GN=GNB2 PE=1 SV=3
KDM4C_HUMAN	37	121958	3	1	2	1	0.05	Lysine-specific demethylase 4C OS=Homo sapiens GN=KDM4C PE=1 SV=2
SCLT1_HUMAN	36	81203	17	3	3	1	0.08	Sodium channel and clathrin linker 1 OS=Homo sapiens GN=SCLT1 PE=1 SV=2

ERGI1_HUMAN	34	32971	3	1	1	1	0.2	Endoplasmic reticulum-Golgi intermediate compartment protein 1 OS=Homo sapiens GN=ERGIC1 PE=1 SV=1
PAR6B_HUMAN	33	41214	5	2	2	1	0.16	Partitioning defective 6 homolog beta OS=Homo sapiens GN=PARD6B PE=1 SV=1
KIF4A_HUMAN	32	141390	20	4	3	1	0.04	Chromosome-associated kinesin KIF4A OS=Homo sapiens GN=KIF4A PE=1 SV=3
MTCH2_HUMAN	31	33936	4	1	2	1	0.19	Mitochondrial carrier homolog 2 OS=Homo sapiens GN=MTCH2 PE=1 SV=1
HEAT6_HUMAN	31	130410	9	4	3	1	0.05	HEAT repeat-containing protein 6 OS=Homo sapiens GN=HEATR6 PE=1 SV=1
KAP1_HUMAN	30	43388	19	1	2	1	0.15	cAMP-dependent protein kinase type I-beta regulatory subunit OS=Homo sapiens GN=PRKAR1B PE=1 SV=4
RGPA2_HUMAN	29	213088	16	2	2	1	0.03	Ral GTPase-activating protein subunit alpha-2 OS=Homo sapiens GN=RALGAPA2 PE=1 SV=2
PCAT2_HUMAN	29	60797	8	1	3	1	0.1	Lysophosphatidylcholine acyltransferase 2 OS=Homo sapiens GN=LPCAT2 PE=1 SV=1
HPSE_HUMAN	28	61395	3	1	2	1	0.1	Heparanase OS=Homo sapiens GN=HPSE PE=1 SV=2
HOOK2_HUMAN	28	83726	13	1	1	1	0.07	Protein Hook homolog 2 OS=Homo sapiens GN=HOOK2 PE=1 SV=3
USH1C_HUMAN	27	62400	2	1	1	1	0.1	Harmonin OS=Homo sapiens GN=USH1C PE=1 SV=3
CDK1_HUMAN	26	34131	15	1	1	1	0.19	Cyclin-dependent kinase 1 OS=Homo sapiens GN=CDK1 PE=1 SV=3

S35E1_HUMAN	26	45086	2	1	1	1	0.14	Solute carrier family 35 member E1 OS=Homo sapiens GN=SLC35E1 PE=1 SV=2
PKDRE_HUMAN	25	257908	148	1	1	1	0.02	Polycystic kidney disease and receptor for egg jelly-related protein OS=Homo sapiens GN=PKDREJ PE=2 SV=2
TRI14_HUMAN	25	50597	3	1	2	1	0.13	Tripartite motif-containing protein 14 OS=Homo sapiens GN=TRIM14 PE=2 SV=2
NOL12_HUMAN	24	24648	20	1	1	1	0.27	Nucleolar protein 12 OS=Homo sapiens GN=NOL12 PE=1 SV=1
LUZP2_HUMAN	23	39275	2	1	2	1	0.16	Leucine zipper protein 2 OS=Homo sapiens GN=LUZP2 PE=2 SV=2
THADA_HUMAN	21	222886	8	1	2	1	0.03	Thyroid adenoma-associated protein OS=Homo sapiens GN=THADA PE=1 SV=1
URFB1_HUMAN	20	160582	5	1	2	1	0.04	UHRF1-binding protein 1 OS=Homo sapiens GN=UHRF1BP1 PE=1 SV=1
VP26A_HUMAN	17	38260	1	1	1	1	0.17	Vacuolar protein sorting-associated protein 26A OS=Homo sapiens GN=VPS26A PE=1 SV=2

Proteins identified in CTR experiment (replicate 1)

Accession	Score	Mass	Matches	Match(sig)	Sequences	Seq(sig)	emPAI	Description
K2C1_HUMAN	3254	66170	86	80	22	21	6.36	Keratin, type II cytoskeletal 1 OS=Homo sapiens GN=KRT1 PE=1 SV=6
K22E_HUMAN	1012	65678	38	31	15	12	2.28	Keratin, type II cytoskeletal 2 epidermal OS=Homo sapiens GN=KRT2 PE=1 SV=2

K2C5_HUMAN	362	62568	16	16	9	9	1.37	Keratin, type II cytoskeletal 5 OS=Homo sapiens GN=KRT5 PE=1 SV=3
K2C6C_HUMAN	304	60273	13	12	7	6	0.82	Keratin, type II cytoskeletal 6C OS=Homo sapiens GN=KRT6C PE=1 SV=3
K2C73_HUMAN	93	59457	6	5	3	2	0.22	Keratin, type II cytoskeletal 73 OS=Homo sapiens GN=KRT73 PE=1 SV=1
K1C10_HUMAN	1875	59020	50	40	16	10	1.76	Keratin, type I cytoskeletal 10 OS=Homo sapiens GN=KRT10 PE=1 SV=6
K1C14_HUMAN	300	51872	18	10	8	5	0.78	Keratin, type I cytoskeletal 14 OS=Homo sapiens GN=KRT14 PE=1 SV=4
K1C9_HUMAN	637	62255	35	27	11	10	1.89	Keratin, type I cytoskeletal 9 OS=Homo sapiens GN=KRT9 PE=1 SV=3
LMAN2_HUMAN	418	40545	24	15	7	5	1.1	Vesicular integral-membrane protein VIP36 OS=Homo sapiens GN=LMAN2 PE=1 SV=1
GBLP_HUMAN	409	35511	16	15	7	7	2.26	Guanine nucleotide-binding protein subunit beta-2-like 1 OS=Homo sapiens GN=GNB2L1 PE=1 SV=3
ATPG_HUMAN	379	33032	14	14	4	4	1.06	ATP synthase subunit gamma, mitochondrial OS=Homo sapiens GN=ATP5C1 PE=1 SV=1
NB5R3_HUMAN	374	34441	21	15	8	6	1.84	NADH-cytochrome b5 reductase 3 OS=Homo sapiens GN=CYB5R3 PE=1 SV=3
PHB2_HUMAN	282	33276	12	11	6	5	1.46	Prohibitin-2 OS=Homo sapiens GN=PHB2 PE=1 SV=2
VDAC1_HUMAN	250	30868	13	9	5	4	1.17	Voltage-dependent anion-selective channel protein 1 OS=Homo sapiens GN=VDAC1 PE=1 SV=2

EF1D_HUMAN	229	31217	13	10	5	4	1.16	Elongation factor 1-delta OS=Homo sapiens GN=EEF1D PE=1 SV=5
SFXN1_HUMAN	211	35881	10	5	5	3	0.65	Sideroflexin-1 OS=Homo sapiens GN=SFXN1 PE=1 SV=4
MTCH2_HUMAN	188	33936	12	8	3	3	0.7	Mitochondrial carrier homolog 2 OS=Homo sapiens GN=MTCH2 PE=1 SV=1
GBB2_HUMAN	180	38048	12	8	3	3	0.6	Guanine nucleotide-binding protein G(I)/G(S)/G(T) subunit beta-2 OS=Homo sapiens GN=GNB2 PE=1 SV=3
OXDD_HUMAN	148	38024	81	12	1	1	0.17	D-aspartate oxidase OS=Homo sapiens GN=DDO PE=2 SV=1
PPT1_HUMAN	142	34627	6	5	2	2	0.41	Palmitoyl-protein thioesterase 1 OS=Homo sapiens GN=PPT1 PE=1 SV=1
LRC59_HUMAN	130	35308	9	7	4	3	0.66	Leucine-rich repeat-containing protein 59 OS=Homo sapiens GN=LRRC59 PE=1 SV=1
RDH11_HUMAN	121	35763	8	2	3	1	0.18	Retinol dehydrogenase 11 OS=Homo sapiens GN=RDH11 PE=1 SV=2
OCAD1_HUMAN	105	27780	2	2	1	1	0.24	OCIA domain-containing protein 1 OS=Homo sapiens GN=OCIAD1 PE=1 SV=1
TMX1_HUMAN	98	32170	9	5	4	3	0.75	Thioredoxin-related transmembrane protein 1 OS=Homo sapiens GN=TMX1 PE=1 SV=1
ERGI1_HUMAN	77	32971	7	7	3	3	0.72	Endoplasmic reticulum-Golgi intermediate compartment protein 1 OS=Homo sapiens GN=ERGIC1 PE=1 SV=1
RS3_HUMAN	70	26842	3	2	2	1	0.25	40S ribosomal protein S3 OS=Homo sapiens GN=RPS3 PE=1 SV=2

SPEE_HUMAN	68	34373	5	4	3	2	0.42	Spermidine synthase OS=Homo sapiens GN=SRM PE=1 SV=1
SNAA_HUMAN	68	33667	4	3	3	2	0.43	Alpha-soluble NSF attachment protein OS=Homo sapiens GN=NAPA PE=1 SV=3
SGTA_HUMAN	67	34270	2	2	1	1	0.19	Small glutamine-rich tetratricopeptide repeat-containing protein alpha OS=Homo sapiens GN=SGTA PE=1 SV=1
CD63_HUMAN	66	26474	2	2	1	1	0.25	CD63 antigen OS=Homo sapiens GN=CD63 PE=1 SV=2
PP2AA_HUMAN	66	36142	3	1	3	1	0.18	Serine/threonine-protein phosphatase 2A catalytic subunit alpha isoform OS=Homo sapiens GN=PPP2CA PE=1 SV=1
RS2_HUMAN	65	31590	4	4	2	2	0.46	40S ribosomal protein S2 OS=Homo sapiens GN=RPS2 PE=1 SV=2
EF1A1_HUMAN	64	50451	4	1	2	1	0.13	Elongation factor 1-alpha 1 OS=Homo sapiens GN=EEF1A1 PE=1 SV=1
LDHA_HUMAN	63	36950	4	3	3	2	0.38	L-lactate dehydrogenase A chain OS=Homo sapiens GN=LDHA PE=1 SV=2
NAIF1_HUMAN	58	35370	8	8	1	1	0.18	Nuclear apoptosis-inducing factor 1 OS=Homo sapiens GN=NAIF1 PE=1 SV=1
SSRA_HUMAN	57	32215	1	1	1	1	0.2	Translocon-associated protein subunit alpha OS=Homo sapiens GN=SSR1 PE=1 SV=3
DCD_HUMAN	57	11391	3	2	2	1	0.68	Dermcidin OS=Homo sapiens GN=DCD PE=1 SV=2
KT3K_HUMAN	52	34618	2	2	1	1	0.19	Ketosamine-3-kinase OS=Homo sapiens GN=FN3KRP PE=1 SV=2

KIF4B_HUMAN	51	141829	25	9	4	1	0.04	Chromosome-associated kinesin KIF4B OS=Homo sapiens GN=KIF4B PE=2 SV=2
ADT2_HUMAN	50	33059	4	2	2	1	0.2	ADP/ATP translocase 2 OS=Homo sapiens GN=SLC25A5 PE=1 SV=7
OTUB1_HUMAN	49	31492	2	1	1	1	0.21	Ubiquitin thioesterase OTUB1 OS=Homo sapiens GN=OTUB1 PE=1 SV=2
PIPNB_HUMAN	48	31805	4	2	3	2	0.46	Phosphatidylinositol transfer protein beta isoform OS=Homo sapiens GN=PITPNB PE=1 SV=2
IPYR2_HUMAN	47	38409	2	1	2	1	0.17	Inorganic pyrophosphatase 2, mitochondrial OS=Homo sapiens GN=PPA2 PE=1 SV=2
DIRA2_HUMAN	47	22813	2	2	1	1	0.3	GTP-binding protein Di-Ras2 OS=Homo sapiens GN=DIRAS2 PE=1 SV=1
ANXA2_HUMAN	46	38808	4	1	3	1	0.17	Annexin A2 OS=Homo sapiens GN=ANXA2 PE=1 SV=2
ZN608_HUMAN	44	163135	3	1	2	1	0.04	Zinc finger protein 608 OS=Homo sapiens GN=ZNF608 PE=1 SV=4
RS3A_HUMAN	44	30154	3	2	2	1	0.22	40S ribosomal protein S3a OS=Homo sapiens GN=RPS3A PE=1 SV=2
MPCP_HUMAN	42	40525	3	3	1	1	0.16	Phosphate carrier protein, mitochondrial OS=Homo sapiens GN=SLC25A3 PE=1 SV=2
UACA_HUMAN	41	163545	25	6	4	1	0.04	Uveal autoantigen with coiled-coil domains and ankyrin repeats OS=Homo sapiens GN=UACA PE=1 SV=2
PGTB2_HUMAN	39	37585	3	2	2	1	0.17	Geranylgeranyl transferase type-2 subunit beta OS=Homo sapiens GN=RABGGTB PE=1 SV=2

RMP_HUMAN	39	60252	11	2	2	1	0.1	Unconventional prefoldin RPB5 interactor 1 OS=Homo sapiens GN=URI1 PE=1 SV=3
DHB11_HUMAN	38	33257	5	1	2	1	0.2	Estradiol 17-beta-dehydrogenase 11 OS=Homo sapiens GN=HSD17B11 PE=1 SV=3
ELAV1_HUMAN	37	36240	1	1	1	1	0.18	ELAV-like protein 1 OS=Homo sapiens GN=ELAVL1 PE=1 SV=2
RLA0_HUMAN	36	34423	2	1	2	1	0.19	60S acidic ribosomal protein P0 OS=Homo sapiens GN=RPLP0 PE=1 SV=1
DECR_HUMAN	35	36330	2	1	2	1	0.18	2,4-dienoyl-CoA reductase, mitochondrial OS=Homo sapiens GN=DECR1 PE=1 SV=1
BACH2_HUMAN	34	94644	21	2	3	1	0.07	Transcription regulator protein BACH2 OS=Homo sapiens GN=BACH2 PE=1 SV=1
MY18A_HUMAN	34	234168	16	1	3	1	0.03	Unconventional myosin-XVIIIa OS=Homo sapiens GN=MYO18A PE=1 SV=3
HYDIN_HUMAN	33	580945	16	2	2	1	0.01	Hydrocephalus-inducing protein homolog OS=Homo sapiens GN=HYDIN PE=1 SV=3
PAR6B_HUMAN	33	41214	3	2	2	1	0.16	Partitioning defective 6 homolog beta OS=Homo sapiens GN=PARD6B PE=1 SV=1
RGPA2_HUMAN	32	213088	16	3	4	1	0.03	Ral GTPase-activating protein subunit alpha-2 OS=Homo sapiens GN=RALGAPA2 PE=1 SV=2
PDXK_HUMAN	32	35308	1	1	1	1	0.19	Pyridoxal kinase OS=Homo sapiens GN=PDXK PE=1 SV=1
CNNM1_HUMAN	32	105426	4	1	3	1	0.06	Metal transporter CNNM1 OS=Homo sapiens GN=CNNM1 PE=2 SV=3
SCLT1_HUMAN	31	81203	10	2	2	1	0.08	Sodium channel and clathrin linker 1 OS=Homo sapiens GN=SCLT1 PE=1 SV=2

LC1L1_HUMAN	30	18078	1	1	1	1	0.39	Putative lipocalin 1-like protein 1 OS=Homo sapiens GN=LCN1P1 PE=5 SV=1
QRIC2_HUMAN	29	181228	15	1	4	1	0.03	Glutamine-rich protein 2 OS=Homo sapiens GN=QRICH2 PE=1 SV=1
CAD24_HUMAN	28	88154	1	1	1	1	0.07	Cadherin-24 OS=Homo sapiens GN=CDH24 PE=1 SV=1
HEAT6_HUMAN	27	130410	8	3	2	1	0.05	HEAT repeat-containing protein 6 OS=Homo sapiens GN=HEATR6 PE=1 SV=1
SREC2_HUMAN	25	96980	4	1	2	1	0.06	Scavenger receptor class F member 2 OS=Homo sapiens GN=SCARF2 PE=1 SV=4
PSDE_HUMAN	25	34726	3	2	2	2	0.41	26S proteasome non-ATPase regulatory subunit 14 OS=Homo sapiens GN=PSMD14 PE=1 SV=1
FPGT_HUMAN	24	67525	5	1	3	1	0.09	Fucose-1-phosphate guanylyltransferase OS=Homo sapiens GN=FPGT PE=1 SV=2
FOLR1_HUMAN	23	30712	1	1	1	1	0.22	Folate receptor alpha OS=Homo sapiens GN=FOLR1 PE=1 SV=3
CRBG3_HUMAN	19	117378	6	1	4	1	0.05	Beta/gamma crystallin domain-containing protein 3 OS=Homo sapiens GN=CRYBG3 PE=2 SV=2
MK07_HUMAN	17	88787	4	1	2	1	0.07	Mitogen-activated protein kinase 7 OS=Homo sapiens GN=MAPK7 PE=1 SV=2
RMXL3_HUMAN	16	115724	4	2	2	1	0.05	RNA-binding motif protein, X-linked-like-3 OS=Homo sapiens GN=RBMXL3 PE=2 SV=2

Proteins identified in CTR experiment (replicate 2)

Note: For each protein the following parameters are reported: Mascot score (Score), molecular weight (mass), number of matched peptides (Matches) and unique peptides used in the identification process (Match(sig)), number of sequences (Sequences) and the number of significant distinct sequence matches in the protein identification process (Seq(sig)) and the relative quantitation of each protein (emPAI).

Figure S5: Mascot Search results for syntaxins identification

MATRIX SCIENCE MASCOT Search Results

Protein View: STX4_HUMAN

Syntaxin-4 OS=Homo sapiens GN=STX4 PE=1 SV=2

Database: SwissProt
Score: 135
Nominal mass (M_r): 34273
Calculated pI: 5.92
Taxonomy: Homo sapiens

Sequence similarity is available as an NCBI BLAST search of STX4_HUMAN against nr.

Search parameters

MS data file: 20160628_cc_ptero_1plh_2.mgf
Enzyme: Trypsin: cuts C-term side of KR unless next residue is P.
Fixed modifications: Carbamidomethyl (C)
Variable modifications: Phospho (ST), Oxidation (M)

Protein sequence coverage: 13%

Matched peptides shown in ***bold red***.

```
  1 MRDRTHELRQ GDDSSDEEDK ERVALVVHPG TARLGSPDEE FFHKVRTIRQ
 51 TIVKLGNKVQ ELEKQQVTIL ATPLPEESMK QELQNLRDEI KQLGREIRLQ
101 LKAIEPQKEE ADENYNSVNT RMRKTQHGVL SQQFVELINK CNSMQSEYRE
151 KNVERIRRQL KITNAGMVSD EELEQMLDSG QSEVFVSNIL KDTQVTRQAL
201 NEISARHSEI QQLERSIREL HDIFTFLATE VEMQGEMINR IEKNILSSAD
251 YVERGQEHVK TALENQKKAR KKKVLIAICV SITVVLLAVI IGVTVVG
```

Unformatted sequence string: 297 residues (for pasting into other applications).

Sort peptides by ◉ Residue Number ○ Increasing Mass ○ Decreasing Mass

Show predicted peptides also

Query	Start - End	Observed	Mr(expt)	Mr(calc)	ppm	M	Score	Expect	Rank	U	Peptide
1021	34 - 44	653.3088	1304.6030	1304.6037	-0.55	0	41	0.0012	1	U	R.LGSPDEEFFHK.V
2587	103 - 121	763.0027	2285.9862	2285.9801	2.69	1	33	0.016	1	U	K.AIEPQKEEADENYNSVNTR.M + Phospho (ST)
2588	103 - 121	763.0027	2285.9862	2285.9801	2.69	1	22	0.11	1	U	K.AIEPQKEEADENYNSVNTR.M + Phospho (ST)
2589	103 - 121	763.0027	2285.9862	2285.9801	2.69	1	31	0.0089	1	U	K.AIEPQKEEADENYNSVNTR.M + Phospho (ST)
2590	103 - 121	1144.0009	2285.9872	2285.9801	3.09	1	18	0.09	1	U	K.AIEPQKEEADENYNSVNTR.M + Phospho (ST)
2591	103 - 121	1144.0009	2285.9872	2285.9801	3.09	1	9	0.62	1	U	K.AIEPQKEEADENYNSVNTR.M + Phospho (ST)
2592	103 - 121	1144.0017	2285.9889	2285.9801	3.84	1	23	0.095	1	U	K.AIEPQKEEADENYNSVNTR.M + Phospho (ST)
952	244 - 254	633.8203	1265.6261	1265.6252	0.73	0	72	2.3e-006	1	U	K.NILSSADYVER.G
953	244 - 254	633.8203	1265.6261	1265.6252	0.73	0	63	1.9e-005	1	U	K.NILSSADYVER.G

MATRIX SCIENCE MASCOT Search Results

Protein View: STX7_HUMAN

Syntaxin-7 OS=Homo sapiens GN=STX7 PE=1 SV=4

**Database:**	SwissProt
**Score:**	147
**Nominal mass (M_r):**	29911
**Calculated pI:**	5.41
**Taxonomy:**	Homo sapiens

Sequence similarity is available as an NCBI BLAST search of STX7_HUMAN against nr.

Search parameters

**MS data file:**	20160628_cc_ptero_1p1h_2.mgf
**Enzyme:**	Trypsin: cuts C-term side of KR unless next residue is P.
**Fixed modifications:**	Carbamidomethyl (C)
**Variable modifications:**	Phospho (ST), Oxidation (M)

Protein sequence coverage: 10%

Matched peptides shown in ***bold red***.

```
  1 MSYTPGVGGD PAQLAQRISS NIQKITQCSV EIQRTLNQLG TPQDSPELRQ
 51 QLQQKQQYTN QLAKETDKYI KEFGSLPTTP SEQRQRKIQK DRLVAEFTTS
101 LTNFQKVQRQ AAEREKEFVA RVRASSRVSG SFPEDSSKER NLVSWESQTQ
151 PQVQVQDEEI TEDDLRLIHE RESSIRQLEA DIMDINEIFK DLGMMIHEQG
201 DVIDSIEANV ENAEVHVQQA NQQLSRAADY QRKSRKTLCI IILILVIGVA
251 IISLIIWGLN H
```

Unformatted sequence string: 261 residues (for pasting into other applications).

Sort peptides by ◉ Residue Number ○ Increasing Mass ○ Decreasing Mass

Show predicted peptides also

Query	Start - End	Observed	Mr(expt)	Mr(calc)	ppm	M	Score	Expect	Rank	U	Peptide
1301	35 - 49	834.9308	1667.8471	1667.8479	-0.43	0	56	1.8e-005	1	U	R.TLNQLGTPQDSPELR.Q
1302	35 - 49	834.9308	1667.8471	1667.8479	-0.43	0	73	7.6e-007	1	U	R.TLNQLGTPQDSPELR.Q
1303	35 - 49	834.9308	1667.8471	1667.8479	-0.43	0	34	0.0017	1	U	R.TLNQLGTPQDSPELR.Q
1304	72 - 84	724.8561	1447.6977	1447.6943	2.35	0	33	0.006	1	U	K.EFGSLPTTPSEQR.Q
1305	72 - 84	724.8561	1447.6977	1447.6943	2.35	0	38	0.0012	1	U	K.EFGSLPTTPSEQR.Q

MATRIX SCIENCE MASCOT Search Results

Protein View: STX12_HUMAN

Syntaxin-12 OS=Homo sapiens GN=STX12 PE=1 SV=1

**Database:**	SwissProt
**Score:**	137
**Nominal mass (M_r):**	31736
**Calculated pI:**	5.45
**Taxonomy:**	Homo sapiens

Sequence similarity is available as an NCBI BLAST search of STX12_HUMAN against nr.

Search parameters

**MS data file:**	20160628_cc_ptero_1plh_2.mgf
**Enzyme:**	Trypsin: cuts C-term side of KR unless next residue is P.
**Fixed modifications:**	Carbamidomethyl (C)
**Variable modifications:**	Phospho (ST), Oxidation (M)

Protein sequence coverage: 10%

Matched peptides shown in ***bold red***.

```
  1 MSYGPLDMYR NPGPSGPQLR DFSSIIQTCS GNIQRISQAT AQIKNLMSQL
 51 GTKQDSSKLQ ENLQQLQHST NQLAKETNEL LKELGSLPLP LSTSEQRQQR
101 LQKERLMNDF SAALNNFQAV QRRVSEKEKE SIARARAGSR LSAEERQREE
151 QLVSFDSHEE WNQMQSQEDE VAITEQDLEL IKERETAIRQ LEADILDVNQ
201 IFKDLAMMIH DQGDLIDSIE ANVESSEVHV ERATEQLQRA AYYQKKSRKK
251 MCILVLVLSV IILILGLIIW LVYKTK
```

Unformatted sequence string: 276 residues (for pasting into other applications).

Sort peptides by ◉ Residue Number ○ Increasing Mass ○ Decreasing Mass

Show predicted peptides also

Query	Start - End	Observed	Mr(expt)	Mr(calc)	ppm	M	Score	Expect	Rank	U	Peptide
1888	21 - 35	863.4171	1724.8196	1724.8152	2.53	0	72	2.3e-006	1	U	R.DFSSIIQTCSGNIQR.I
1889	21 - 35	863.4171	1724.8196	1724.8152	2.53	0	78	6e-007	1	U	R.DFSSIIQTCSGNIQR.I
1739	83 - 97	813.9475	1625.8805	1625.8624	11.1	0	22	0.0081	1	U	K.ELGSLPLPLSTSEQR.Q
1740	83 - 97	813.9475	1625.8805	1625.8624	11.1	0	32	0.0032	1	U	K.ELGSLPLPLSTSEQR.Q

MATRIX SCIENCE MASCOT Search Results

Protein View: STX6_HUMAN

Syntaxin-6 OS=Homo sapiens GN=STX6 PE=1 SV=1

Database: SwissProt
Score: 99
Nominal mass (M_r): 29215
Calculated pI: 4.84
Taxonomy: Homo sapiens

Sequence similarity is available as an NCBI BLAST search of STX6_HUMAN against nr.

Search parameters

MS data file: 20160628_cc_ptero_1plh_2.mgf
Enzyme: Trypsin: cuts C-term side of KR unless next residue is P.
Fixed modifications: Carbamidomethyl (C)
Variable modifications: Phospho (ST), Oxidation (M)

Protein sequence coverage: 9%

Matched peptides shown in ***bold red***.

```
  1 MSMEDPFFVV KGEVQKAVNT AQGLFQRWTE LLQDPSTATR EEIDWTTNEL
 51 RNNLRSIEWD LEDLDETISI VEANPRKFNL DATELSIRKA FITSTRQVVR
101 DMKDQMSTSS VQALAERKNR QALLGDSGSQ NWSTGTTDKY GRLDRELQRA
151 NSHFIEEQQA QQQLIVEQQD EQLELVSGSI GVLKNMSQRI GGELEEQAVM
201 LEDFSHELES TQSRLDNVMK KLAKVSHMTS DRRQWCAIAI LFAVLLVVLI
251 LFLVL
```

Unformatted sequence string: 255 residues (for pasting into other applications).

Sort peptides by ◉ Residue Number ○ Increasing Mass ○ Decreasing Mass

Show predicted peptides also

Query	Start - End	Observed	Mr(expt)	Mr(calc)	ppm	M	Score	Expect	Rank	U	Peptide
753	17 - 27	602.8257	1203.6369	1203.6360	0.77	0	52	0.00017	1	U	K.AVNTAQGLFQR.W
754	17 - 27	602.8257	1203.6369	1203.6360	0.77	0	65	9.3e-006	1	U	K.AVNTAQGLFQR.W
1473	28 - 40	759.3825	1516.7505	1516.7522	-1.12	0	33	0.007	1	U	R.WTELLQDPSTATR.E

Biological assays

Figure S6: AlphaScreeen assay: GST-Acceptor beads, Streptavidin- Donor beads and a GST-Biotin bivalent ligand were incubated in absence (CTR) and in presence of PTS at two different concentrations.

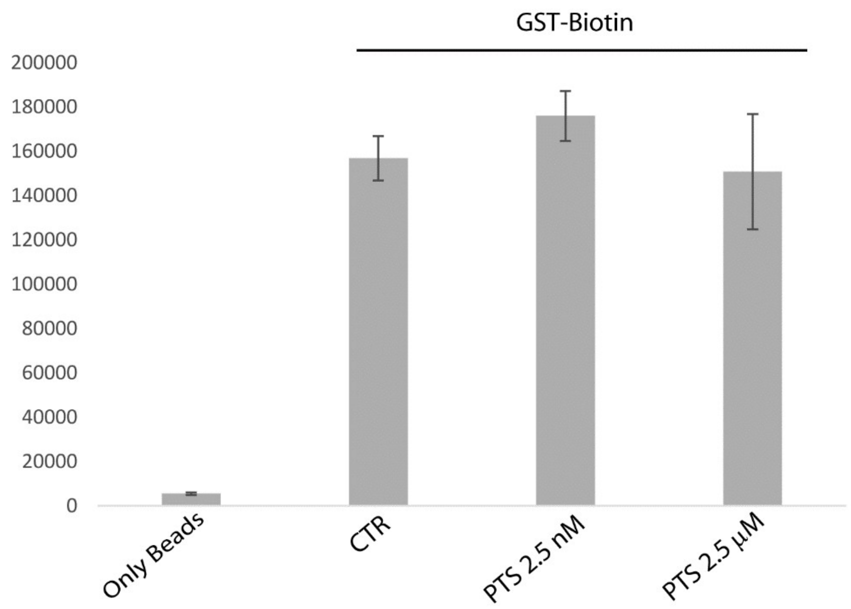

Figure S7: Evaluation of PTS cytotoxic effects were carried out by MTT cell viability assays (at different PTS concentrations, 24h incubation).

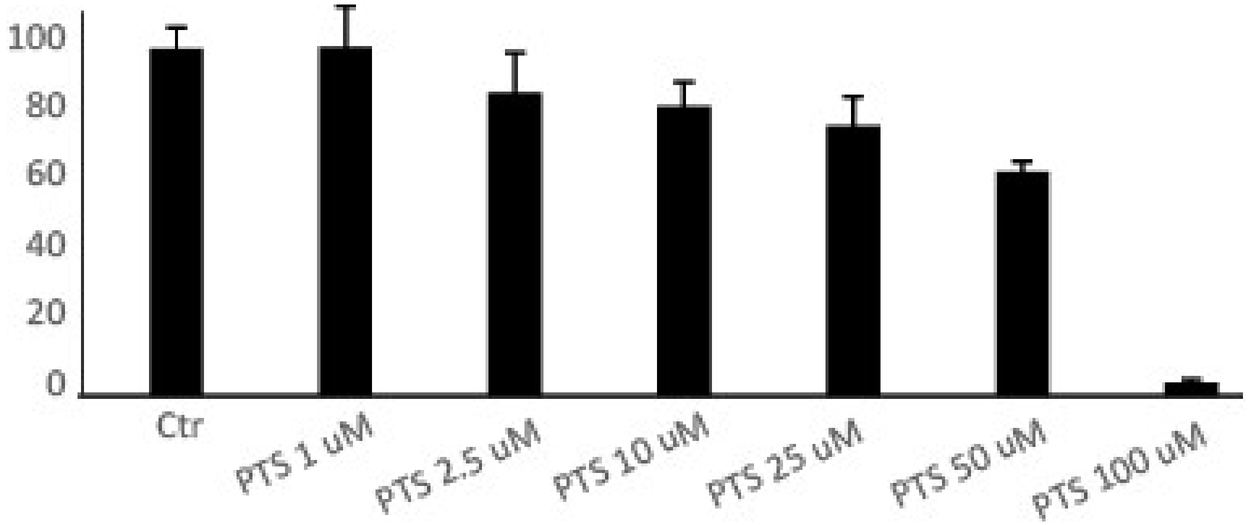

Figure S8: STRING analysis of all the putative PTS partners arranged on the basis of their biological functions.

As reported by McCormack et al. 2013, PTS is well known for its wide biological potential. Its antioxidant activity could be due to PTS interaction with several proteins, such as thioredoxin related transmembrane protein 2 (TMX2), mitochondrial NADH:ubiquinone oxidoreductase complex (NDUFAF1), dehydrogenase/reductase SDR family member 1 (DHRS1), NADH-cytochrome b5 reductase 1 (CYB5R1). Furthermore, theING same authors regognized PTS to ameliorate diabetes, and this could match with its action on those proteins involved in vesicles exocytosis as syntaxins (STX), synaptophysin-like protein 1 (SYPL1), and secretory carrier-associated membrane protein 3 (SCAMP3). Finally, PTS is well known to act on metabolic diseases, and this could match with its interaction with those proteins involved in FA metabolism, such as very-long-chain 3-oxoacyl-CoA reductase (HSD17B12), ubiquinone biosynthesis protein COQ9 (COQ9), delta(3,5)-delta(2,4)-dienoyl-CoA isomerase, (ECH1), and sphingolipid delta(4)-desaturase DES1 (DGS1).

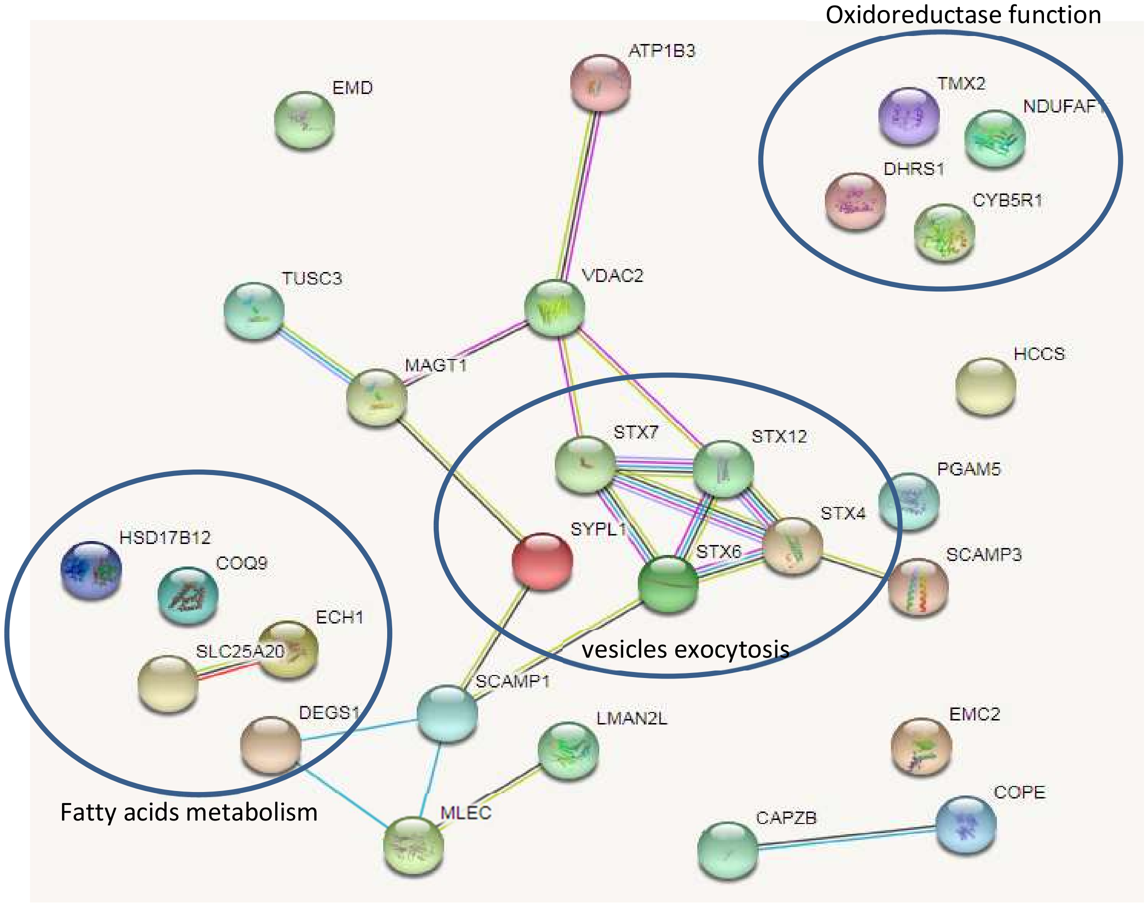

Supplement: Supplementary file 1 [file molecules-25-02885-s001.pdf]
